# Guideline on the use of iodinated contrast media in patients with kidney disease 2018

**DOI:** 10.1007/s10157-019-01750-5

**Published:** 2019-11-11

**Authors:** Yoshitaka Isaka, Hiromitsu Hayashi, Kazutaka Aonuma, Masaru Horio, Yoshio Terada, Kent Doi, Yoshihide Fujigaki, Hideo Yasuda, Taichi Sato, Tomoyuki Fujikura, Ryohei Kuwatsuru, Hiroshi Toei, Ryusuke Murakami, Yoshihiko Saito, Atsushi Hirayama, Toyoaki Murohara, Akira Sato, Hideki Ishii, Tadateru Takayama, Makoto Watanabe, Kazuo Awai, Seitaro Oda, Takamichi Murakami, Yukinobu Yagyu, Nobuhiko Joki, Yasuhiro Komatsu, Takamasa Miyauchi, Yugo Ito, Ryo Miyazawa, Yoshihiko Kanno, Tomonari Ogawa, Hiroki Hayashi, Eri Koshi, Tomoki Kosugi, Yoshinari Yasuda, Japanese Society of Nephrology, Japan Radiological Society, and Japanese Circulation Society Joint Working Group

**Affiliations:** 1 Department of Nephrology, Osaka University Graduate School of Medicine, Osaka, Japan; 2 Department of Clinical Radiology, Graduate School of Medicine, Nippon Medical School, Tokyo, Japan; 3 Cardiology Department, Institute of Clinical Medicine, University of Tsukuba, Ibaraki, Japan; 4 Kansai Medical Hospital, Osaka, Japan; 5 Department of Endocrinology, Metabolism and Nephrology, Kochi Medical School, Kochi University, Kochi, Japan; 6 Department of Acute Medicine, The University of Tokyo, Tokyo, Japan; 7 Division of Nephrology, Department of Internal Medicine, Teikyo University School of Medicine, Tokyo, Japan; 8 First Department of Medicine, Hamamatsu University School of Medicine, Shizuoka, Japan; 9 Department of Radiology, Graduate School of Medicine, Juntendo University, Tokyo, Japan; 10 Department of Cardiovascular Medicine, Nara Medical University, Nara, Japan; 11 Department of Cardiology, Osaka Police Hospital, Osaka, Japan; 12 Department of Cardiology, Nagoya University Graduate School of Medicine, Aichi, Japan; 13 Department of Cardiology, Faculty of Medicine, University of Tsukuba, Ibaraki, Japan; 14 Division of General Medicine, Department of Medicine, Nihon University School of Medicine, Tokyo, Japan; 15 Department of Diagnostic Radiology, Graduate School of Biomedical and Health Sciences, Hiroshima University, Hiroshima, Japan; 16 Department of Diagnostic Radiology, Faculty of Life Sciences, Kumamoto University, Kumamoto, Japan; 17 Department of Radiology, Kobe University Graduate School of Medicine, Hyogo, Japan; 18 Department of Radiology, Faculty of Medicine, Kindai University, Osaka, Japan; 19 Division of Nephrology, Toho University Ohashi Medical Center, Tokyo, Japan; 20 Department of Healthcare Quality and Safety, Gunma University Graduate School of Medicine, Gunma, Japan; 21 Cedars Sinai Medical Hospital, Los Angeles, CA USA; 22 Department of Nephrology, St. Luke’s International Hospital, Tokyo, Japan; 23 Department of Radiology, St. Luke’s International Hospital, Tokyo, Japan; 24 Department of Nephrology, Tokyo Medical University, Tokyo, Japan; 25 Department of Nephrology and Hypertension, Saitama Medical Center, Saitama, Japan; 26 Department of Nephrology, Fujita Health University School of Medicine, Aichi, Japan; 27 Department of Nephrology, Komaki City Hospital, Aichi, Japan; 28 Nephrology, Nagoya University Graduate School of Medicine, Aichi, Japan; 29 Department of CKD Initiatives/Nephrology, Nagoya University Graduate School of Medicine, Aichi, Japan

## 1. Outline of the revised version of the guideline on the use of iodinated contrast media in patients with kidney disease

### 1.1 Purpose of the revision of the guideline

Diagnostic imaging using iodinated contrast media is an essential procedure in the clinical setting and provides a large amount of beneficial information. However, the use of iodinated contrast media may cause contrast-induced nephropathy (CIN) in patients with chronic kidney disease (CKD). Therefore, the Japan Radiological Society (JRS), the Japanese Circulation Society (JCS), and the Japanese Society of Nephrology (JSN) collaborated and published a guideline on the use of iodinated contrast media in patients with kidney disease (CIN guideline 2012).

The aim of the CIN guideline 2012 was to ensure the prevention of kidney injury induced by iodinated contrast media by promoting the appropriate use of contrast media and the standardization of kidney function testing in patients undergoing contrast radiography. The target audience of the guideline included physicians using contrast media and physicians ordering contrast radiography, as well as other healthcare professionals such as radiation technologists and nurses involved in contrast radiography.

The CIN guideline 2012 was prepared based on articles published during the period from 1960 to the end of August 2011, according to the recommendation of the Medical Information Network Distribution Service (Minds). Therefore, at the time of the publication of the CIN guideline 2012, the diagnostic criteria of acute kidney injury (AKI) was not unified, and both the RIFLE (risk, injury, failure, loss of kidney function, and end-stage renal failure) and Acute Kidney Injury Network (AKIN) definitions were stated. In addition, the CIN guideline 2012 did not comply with the Kidney Disease Improving Global Outcomes (KDIGO) AKI diagnostic criteria. Since the publication of the CIN guideline 2012, and a number of novel research findings have been reported. The European Society of Urogenital Radiology (ESUR) published the 3rd edition of their guideline in 2014, and the American College of Radiology (ACR) published version 10.2 of their guideline in 2016. Therefore, we decided to revise the CIN guideline 2012 in accordance with three academic societies. Meanwhile, although the AKI guideline 2016 was published in compliance with KDIGO’s AKI diagnostic criteria, most papers on CIN do not necessarily conform to them. In our revised CIN guideline, we changed the clinical question (CQ) on the diagnosis of CIN to “How should CIN be diagnosed?” and followed the diagnostic criteria of the previous guideline (“CIN is defined as an increase in serum creatinine (SCr) levels by ≥ 0.5 mg/dL or ≥ 25% from baseline within 72 h after a contrast radiography using iodinated contrast media.”), but decided to include KDIGO’s AKI diagnostic criteria. Future research is necessary to determine whether the AKI diagnostic criteria can be applied to the diagnosis of CIN. Since the ESUR guideline 2018 was available after the last meeting of the committee on February 18, 2017, the committee reconfirmed their consistency with the revision of this guideline.

### 1.2 A cautionary note on the use of the present guideline

The revised guideline has been prepared for use according to the National Health Insurance (NHI) regulations in Japan. The revised guideline provides direction on using contrast media in the clinical setting. Physicians have the final responsibility to maximize the benefits for their patients by deciding, on the basis of their patients’ physical and pathological conditions, whether contrast media should be administered and whether measures to prevent CIN are necessary. Any use of contrast media that is not consistent with the revised guideline reflects the decisions made by the attending physicians on the basis of conditions specific to their patients, and their decisions should be prioritized. The present guideline does not provide any legal basis for prosecuting physicians who do not use contrast media according to the guideline.

### 1.3 Selection of literature, levels of evidence, and grades of recommendations

The revised guideline was prepared according to the procedures proposed by Minds. The guideline writing committee discussed and revised CQs on 9 themes regarding CIN.

The working groups addressed the CQs by critically reviewing literature published from September 1, 2011 to March 31, 2017 in major literature databases (e.g., PubMed, MEDLINE, the Cochrane Library, and the Japan Centra Revuo Medicina [Ichushi]), in addition to the literature referenced in the CIN guideline 2012. Literature published since April 2017 was also included as deemed necessary by the guideline committee. Since the CIN guideline 2012 was prepared according to the Minds guideline 2007, CQs included in 2012 were revised according to the Minds guideline 2007. New CQs added in this revised guideline, CQ3-12, CQ5-6, CQ5-7, and CQ6-4, were prepared according to “Minds Guidebook for Guideline Development 2014” and “Minds Manual for Guideline Development 2017”. Thus, in this revised guideline, 2 kinds of evidence and recommendation evaluation methods were adopted.

A level of evidence and grade of recommendation were assigned to the answers to the CQs.

The levels of evidence and grades of recommendation according to Minds 2007 are as follows:

Level of evidence

-  Level I: Data obtained from a systematic review or meta-analysis of randomized clinical trials
-  Level II: Data obtained from at least one randomized comparative clinical trial
-  Level III: Data obtained from non-randomized comparative clinical trials
-  Level IVa: Cohort studies
-  Level IVb: Case–control or cross-sectional studies
-  Level V: Case reports or case series
-  Level VI: Opinions of special committees or specialists with no basis of patient data

Grade of recommendation

-  Grade A: A given treatment or procedure is recommended based on robust scientific evidence
-  Grade B: A given treatment or procedure is suggested based on scientific evidence
-  Grade C1: A given treatment or procedure may (/might) be considered, although scientific evidence is not available
-  Grade C2: A given treatment or procedure may (/might) be not considered because scientific evidence is not available
-  Grade D: A given treatment or procedure is not recommended because scientific evidence indicating the inefficacy or harm of the treatment/procedure is available

The Delphi method was used to finalize the answer to each CQ and determine its grade of recommendation. The reader should give a higher priority to the grade of recommendation of the answer than to the level of evidence. The grade of recommendation has been decided not only based on the level of evidence, but also on the quality and clinical significance of the evidence, extent, and conclusions of data on harmful effects and cost-effectiveness, depth of coverage by the NHI system, and availability in Japan.

The levels of evidence and grades of recommendation by Minds 2017 are as follows:

Quality of evidence

-  A Strong: Strongly confident
-  B: Medium: Medium confidence
-  C: Weak: Confidence is limited
-  D: Very weak: Almost not confident

Strength of recommendation

-  1: Strongly recommended
-  2: Weakly recommended (suggested)
-  None: Clear recommendations cannot be made

### 1.4 Independent assessment

The present guideline was reviewed by an independent assessment committee consisting of 3 representatives each from the JSN, JRS, and JCS. The final draft of the guideline was published on the websites of the 3 societies along with a request for public comments. The guideline writing committee discussed the comments, used them to revise the guideline when appropriate, and finalized the guideline.

### 1.5 Future plans

Following publication as a printed book from Tokyo Igakusha, the Japanese version of the guideline will be published in the Japanese Journal of Nephrology, as a JCS guideline document, and online on the websites of the member societies. An English version will be prepared and published in the English-language journals of the member societies. The guideline will also be published by the Medical Information Network Distribution Service (Minds) of the Japan Council for Quality Health Care. The full and digest versions of the guideline are planned to be revised every 5 years. A new writing committee will be established by representatives of the member societies to maintain unbiased, appropriate guidelines.

### 1.6 Conflict of interest

The transportation expenses of the committee members were covered by the JSN, JRS, and JCS. Conflict of interest statements were provided by all committee members involved in the preparation or review of the guideline and managed by the relevant societies.

## 2 Diagnosis of contrast-induced nephropathy (CIN)

### 2.1 **CQ 2-1** How CIN should be diagnosed?

Answer:

CIN is diagnosed when serum creatinine (SCr) levels increase by ≥ 0.5 mg/dL or ≥ 25% from baseline within 72 h after contrast radiography using iodinated contrast media. Since CIN is a form of acute kidney injury (AKI), it is also evaluated using the diagnostic criteria of AKI (KDIGO Clinical Practice Guideline for Acute Kidney Injury; KDIGO AKI guideline [1]). According to the diagnostic criteria of the KDIGO AKI guideline, when SCr levels increase by ≥ 0.3 mg/dL within 48 h or ≥ 1.5-fold from the basal level, which is either known or presumed to have occurred within the prior 7 days, or the urine volume decreases to < 0.5 mL/kg/h over 6 h after the administration of the iodinated contrast media, CIN is diagnosed. The severity (stage) of AKI should also be evaluated [1].

**Rationale CQ 2-1**

Since the risk of developing CIN increases as kidney function decreases, it is important to evaluate kidney function on the basis of the latest SCr levels prior to contrast radiography. According to the classification of the severity of CKD, which is determined based on the cause, glomerular filtration rate (GFR), and presence and severity of albuminuria [1], patients with a GFR < 60 mL/min/1.73 m^2^ (G3a-G5) are considered to have CKD in this guideline. Although CKD is also diagnosed in patients with a GFR ≥ 60 mL/min/1.73 m^2^ and albuminuria, only patients with a GFR of < 60 mL/min/1.73 m^2^ are defined as having CKD in the present guideline. The following formula is used to calculate estimated GFR (eGFR).
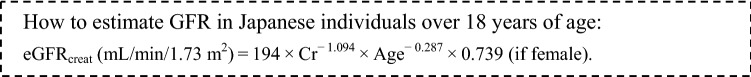


Evaluation of renal function before contrast radiography is performed by estimated GFR (eGFR), but a diagnosis of CIN should be evaluated not by a change in eGFR, but by a change in SCr. Even if GFR decreases due to AKI, SCr increases with a delay of about 24–48 h; therefore, we consider that eGFR calculated based on SCr does not represent the actual GFR in real time.

CIN is a form of AKI that occurs after exposure to iodinated contrast media and is diagnosed on the basis of decreased kidney function after contrast radiography after other causes such as cholesterol embolism have been ruled out. AKI due to CIN is generally reversible. Usually, SCr levels increase to a peak at 3-5 days after onset and return to normal in 7-14 days. However, kidney injury may worsen to the point that hemodialysis is required in some patients.

The criteria for the diagnosis of CIN used in the clinical research of this condition vary among studies. The minimum increase in SCr levels used to define CIN includes 0.5 mg/dL, 1.0 mg/dL, and 25% or 50% from baseline, while the duration of monitoring for CIN included 24 h, 48 h, 72 h, 4 days, and 7 days after contrast radiography. The most commonly used criterion for CIN in clinical research is an increase in SCr levels by ≥ 0.5 mg/dL or ≥ 25% from baseline within 72 h after contrast radiography. However, physicians in the clinical setting should not wait for 72 h and should start closely monitoring the SCr levels from an early stage when CIN is suspected.

The incidence of CIN depends greatly on the diagnostic criteria, and the clinical characteristics of the CIN onset group such as renal function before contrast radiography are also influenced by the diagnostic criteria. The diagnostic criteria of “an increase in SCr levels by ≥ 0.5 mg/dL or ≥ 25% from baseline within 72 h after a contrast radiography using iodinated contrast media” published by the ESUR in 1999 have been widely used [2]. Meanwhile, since CIN is a form of AKI, evaluation of CIN using AKI diagnostic criteria has been attempted. The international diagnostic criteria of AKI include the RIFLE classification created by the Acute Dialysis Quality Initiative (ADQI) in 2004, the AKIN classification created in 2007, and the KDIGO AKI guidelines established in 2012 (KDIGO Clinical Practice Guideline for Acute Kidney Injury) [1]. According to the KDIGO AKI guidelines, the diagnostic criteria of AKI are an increase in SCr by ≥ 0.3 mg/dL within 48 h or a urine volume reduction to < 0.5 mL/kg/h over 6 h. According to KDIGO policy, CIN is a form of AKI and should be evaluated on the same basis. Although the criteria for diagnosing AKI by KDIGO are also supported by the AKI clinical practice guideline 2016 in Japan (AKI guideline 2016) [3], the diagnostic criteria for CIN were not included in AKI guideline 2016. According to the ESUR guideline published in 2018, as a definition of CIN, an increase in SCr by ≥ 0.3 mg/dL from the baseline level within 48–72 h after administration of the contrast medium or a 1.5–1.9-fold increase. However, the onset of CIN is rare in patients with normal renal function and occurs frequently in those with reduced renal function. CIN with oliguria is also rare. A changes in the SCr level of 0.3 mg/dL may be too sharp as a diagnostic criterion for CIN in patients with impaired renal function. Standardization of the diagnostic criteria is necessary to promote clinical research on the prevention of CIN onset, etc. Although the diagnostic criteria of AKI may not be sufficiently useful, the criteria for AKI are widely accepted as applicable to CIN. Therefore, in this revised guideline, both the conventional CIN diagnostic criteria and KDIGO AKI criteria are listed.

**Definition and severity classification of acute kidney injury**

In 2012 KDIGO created the KDIGO AKI guideline. AKI is defined as any of the following: an increase in SCr by ≥ 0.3 mg/dL within 48 h, an increase in SCr to ≥ 1.5 times the baseline, which is known or presumed to have occurred within the prior 7 days, or a urine volume decrease to < 0.5 mL/kg/h for 6 h. The severity (stage) of AKI is defined according to the following criteria.

**Changes in serum Cr**

Renal function is not constant and changes in response to diet, exercise, and body fluid volume changes. In addition, drugs that inhibit the tubular secretion of creatinine increase the SCr level. When ingesting cooked meat or supplements containing creatinine, an increase in SCr levels due to the absorption of creatinine is observed. Therefore, the following points regarding fluctuations in SCr level should be noted:

1. SCr value has a diurnal variation of about 10%.
2. SCr value rises during severe exercise or a large intake of meat and falls when protein intake is restricted.
3. Cimetidine and trimethoprim reduce creatinine excretion via the renal tubules and may raise the SCr level.

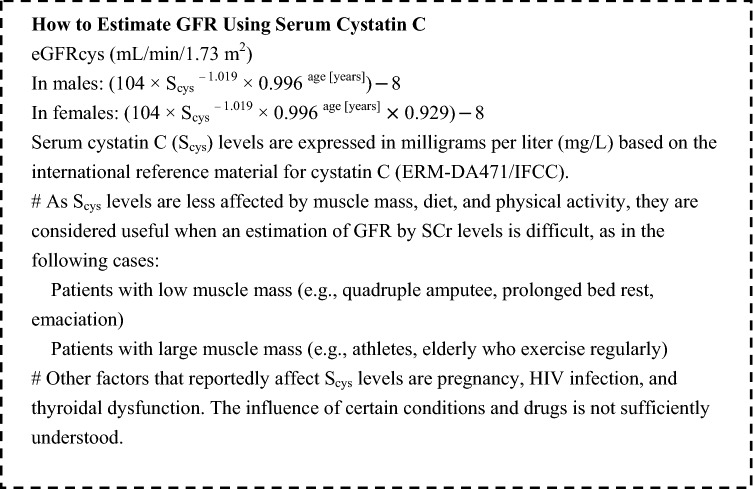


**AKI clinical practice guideline 2016**

The prevalence of acute kidney injury (AKI) is rapidly increasing in the United States at a rate of about 5 times over 20 years due to an aging population and increased incidences of CKD and diabetes mellitus. Since the KDIGO AKI diagnostic criteria were published in 2012 [1], they have been used as the international standard, and the Japanese guidelines for AKI published in 2016 support the KDIGO criteria [3]. AKI represents a broad disease spectrum that develops in the context of various disease states. CIN is one of the most frequent etiologies of AKI, and special attention is needed for high-risk groups such as CKD patients. In terms of evaluating AKI risk, the AKI guideline 2016 summarizes the risks associated with surgical and other clinical conditions such as heart failure that have been shown to be affected by the presence of renal function insufficiency and aging. For sepsis in particular, the frequency of AKI onset is higher in patients taking renin-angiotensin system (RAS) inhibitors.

The SCr level is currently used as a diagnostic marker, and it often rises 24–48 h after surgery. We note that diagnosis by SCr tends to be delayed because of its kinetics, and urinary neutrophil gelatinase-associated lipocalin (NGAL), liver-type fatty acid-binding protein (L-FABP), and urinary cystatin C can be used as a biomarker for early diagnosis. In addition, long-term follow-up of AKI patients is important. In recent years, many clinical studies reported that the long-term prognosis of AKI is poor, and a certain portion of AKI survivors have been known to develop CKD. It has also been shown that they are likely to develop hypertension and heart disease. It is suggested to follow the patients according to their condition for 3 months after the onset of AKI.

It is clear that aging and renal dysfunction are significant risk factors for AKI, and therefore the elderly are a high-risk group. Risk factors for AKI in the elderly include taking RAS inhibitors, diuretics, nonsteroidal anti-inflammatory drugs, vitamin D, and others. In addition, the elderly may easily develop AKI in the context of dehydration, fever, and infection. To facilitate the early detection of AKI and prevention of its exacerbation, it is necessary to minimize the use of drugs that are known to increase risk and to monitor the renal function of elderly patients who regularly take such drugs. When using contrast media in the elderly, we should be aware of the potential for many risk factors for AKI and the need for careful monitoring.

## 3 Risk factors and patient assessment

### 3.1 **CQ3-1** Does the risk of developing CIN increase in CKD patients?

Answer:

CKD (eGFR < 60 mL/min/1.73 m^2^) is a risk factor for the development of CIN. However, the risk depends on the administration route of the contrast media and the pathophysiological condition of the patient (see Chapters 3–6).

**Level of Evidence: IVa/Grade of Recommendation: Not applicable**

### 3. 2 **CQ 3-2** Does aging increase the risk of developing CIN?

Answer:

Aging is a risk factor for the development of CIN.

**Level of Evidence: IVa/Grade of Recommendation: Not applicable**

### 3.3 **CQ 3-3** Does diabetes mellitus increase the risk of developing CIN?

Answer:

Although diabetes mellitus associated with CKD (eGFR < 60 mL/min/1.73 m^2^) is a risk factor for the development of CIN, it is unclear whether diabetes mellitus without CKD is a risk factor.

**Level of Evidence: IVa/Grade of Recommendation: Not applicable**

**Rationale CQ3-1**

In recent years, renal function has been evaluated using GFR instead of the conventional SCr level. As a result, CIN guidelines in each country that were revised after 2014 include the evaluation of renal function by GFR, and it is stated that a decline in GFR is a risk factor for CIN. However, the guidelines differ with respect to the threshold of declined renal function that is considered a risk (Table 1), and the degree of renal dysfunction (GFR) corresponding to avoidance of the use of contrast media does not match among the guidelines. In addition, there have been reports that intravenously administered-iodinated contrast media carries a lower risk of developing CIN than intra-arterial administration, and the risk of developing CIN by intravenous administration of iodinated contrast media is considered lower than previously thought. In the ESUR guideline 2014, eGFR < 45 mL/min/1.73 m^2^ before intravenous contrast media administration and eGFR < 60 mL/min/1.73 m^2^ before intra-arterial contrast media administration are considered to represent a CIN risk, whereas the RANZCR 2016 guideline indicates that the risk of intravenous contrast media-related CIN is likely to be nonexistent for patients with an eGFR > 45 mL/min/1.73 m^2^, and the ACR 2017 indicates that an eGFR < 30 mL/min/1.73 m^2^ is associated with an increased risk of CIN with intravenous injection. In this guideline, we continue to regard CKD (eGFR < 60 mL/min/1.73 m^2^) as a risk factor for CIN, referring to CQ 5-1, 4, 7 for cases of arterial injection and CQ 6-1 for cases of intravenous injection.

**Table 1:** Guidelines for the use of contrast media on each country

	Guideline	ACR 2017 (American College of Radiology)	RANZCR 2016 (The Royal Australian and New Zealand College of Radiologists)	ESUR 2014 (European Society of Urogenital Radiology)	NICE 2014 (The National Institute for Health and Care Excellence)	KDIGO 2012 (Kidney Disease Improving Global Outcomes)	CAR 2011 (Canadian Association of Radiologists)
CQ3-1	Renal function	There is no agreed-upon threshold of serum creatinine elevation or eGFR decrease beyond which the risk of CIN is considered so great that intravascular iodinated contrast medium should never be administered There is very little evidence that IV iodinated contrast material is an independent risk factor for AKI in patients with an eGFR ≥ 30 mL/min/1.73 m^2^. Therefore, if any threshold for CIN risk is to be used, 30 mL/min/1.73 m^2^ seems to be the one with the greatest level of evidence	The risk of intravenous contrast media-related CIN is likely to be non-existent for patients with an eGFR greater than 45 mL/min/1.73 m^2^ The risk of intravenous CIN is also very likely to be low or non-existent for patients with an eGFR 30–45 mL/min/1.73 m^2^ In patients with severe renal function impairment (eGFR less than 30 mL/min/1.73 m^2^) or actively deteriorating renal function (AKI), careful weighing of the risk versus the benefit of iodinated contrast media administration needs to be undertaken	An eGFR < 60 mL/min/1.73 m^2^ before intra-arterial contrast media administration and an eGFR < 45 mL/min/1.73 m^2^ before intravenous contrast media administration are considered to represent a high CIN risk	CKD, especially cases with an eGFR < 40 mL/min/1.73 m^2^, is a risk factor for CIN	The contrast-induced AKI Consensus Working Panel recommended that precautions to reduce the risk should be implemented in patients with a baseline eGFR of 60 mL/min/1.73 m^2^ In light of more recent information, this threshold could probably be lowered to 45 mL/min/1.73 m^2^	eGFR ≥ 60 mL/min: very low risk of CIN. These patients require no specific prophylaxis or follow-up eGFR 45–59 mL/min: low risk of CIN. In the absence of additional risk factors, patients receiving IV contrast media require no specific prophylaxis or follow-up. For patients receiving intra-arterial contrast media, preventative measures are recommended eGFR < 45 mL/min: moderate risk of CIN, preventive measures are recommended. IV hydration is recommended for patients receiving intra-arterial contrast. For intravenous administration, either oral or IV hydration could be used; IV hydration is preferred if the eGFR is < 30 mL/min
CQ3-2	Aging	Risk at age > 60 years	Age should not be considered an independent risk factor	Risk at age > 70 years	Risk at age ≥ 75 years	Risk factor	Risk at age > 70 years
CQ3-3	Diabetes mellitus	Risk factor		Risk factor	Risk in diabetes but only with CKD	Although there is doubt that diabetes by itself is an independent risk factor, in a patient with CKD, it acts as a risk multiplier	Risk factor
	Reference	ACR Manual on Contrast Media Version 10.3	Iondinate Contrast Media Guideline	ESUR Guidelines on Contrast Media 9.0	Acute Kidney Injury: prevention detection and management	KDIGO Clinical Practice Guideline for Acute Kidney Injury. Kidney Int Suppl 2012;2:1–138	Consensus Guidelines for the Prevention of Contrast Induced Nephropathy. Can Assoc Radiol J 2014;65:96–105
		https://www.acr.org/Clinical-Resources/Contrast-Manual	https://www.ranzcr.com/fellows/clinical-radiology/professional-documents/iodinated-contrast-media-guideline-2016-recommendations	http://www.esur.org/esur-guidelines/	https://www.nice.org.uk/Guidance/CG169	http://kdigo.org/guidelines/acute-kidney-injury/	


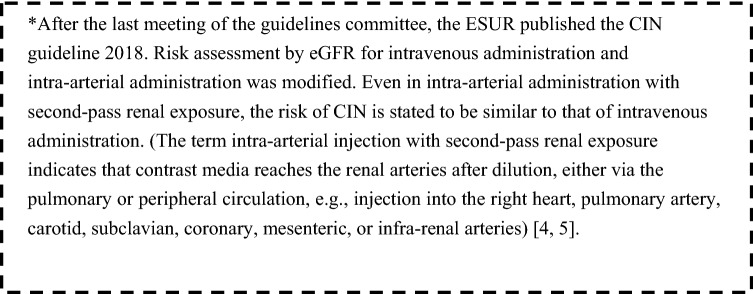


**Rationale CQ3-2 to 3-3**

Many studies have reported that aging and diabetes mellitus may increase the risk of the development of CIN. In a cohort study of 3036 patients with baseline SCr levels (< 1.5 mg/dL) who did not receive prophylaxis while undergoing percutaneous coronary intervention (PCI), CIN occurred in 7.3% of patients [6]. Risk factors for CIN included age (odds ratio [OR] 6.4, 95% confidence interval [CI] 1.01–13.3), female sex (OR 2.0, 95% CI 1.5–2.7), an abnormal left ventricular ejection fraction (LVEF) of < 50% (OR 1.02, 95% CI 1.01–1.04), the presence of anemia with hemoglobin levels of < 11 mg/dL (OR 1.5, 95% CI 1.01–2.4), and systolic hypotension with blood pressure < 100 mmHg (OR 1.5, 95% CI 1.01–2.2). Patients with diabetes mellitus who were receiving insulin therapy were at the highest risk compared with similar patients who were prescribed oral anti-hyperglycemic agents and diet control [6].

In an observational study, CIN developed in 15.44% of 136 patients who underwent CAG and measures to prevent CIN. The risk factors that seemed to display the greatest correlation with the risk of CIN were advanced age and heart failure (LVEF < 40%). The concomitant presence of heart failure, anemia, diabetes mellitus, previous myocardial infarction, and advanced age (> 70 years) was associated with a threefold increased risk of CIN [7]. In the cohort study of 364 patients undergoing arteriography, the frequency of CIN was 7.1%. Although most patients recovered, 1.4% required dialysis. Preexisting renal disease, advanced age, volume of contrast medium, type of study performed (abdominal arteriography), diabetes mellitus, and coexisting heart disease have been reported as risk factors for CIN [8]. According to a review reported in 2007, the classic risk factors for CIN were preexisting renal failure, diabetes mellitus, advanced age, nephrotoxic agent administration, hypovolemia, use of a large amount of contrast medium, ionic hyperosmolar contrast media, and congestive heart failure. Metabolic syndrome, prediabetes, and hyperuricemia have been identified as new risk factors for CIN [9]. CKD (eGFR < 60 mL/min/1.73 m^2^) in diabetic patients is reported to be a risk factor for CIN. However, diabetes mellitus is not necessarily a risk factor for CIN but is a risk-increasing factor. That is, the risk of developing CIN increases in CKD patients with diabetes mellitus [10].

In observational studies investigating whether CKD was involved in the prognosis of renal events in diabetic patients who received PCI, CIN after PCI occurred in 15% of patients in the non-CKD group and 27% in the CKD group, and 0.1% and 3.1% required dialysis in the non-CKD and CKD groups, respectively. The prognostic factors for CIN were associated with low blood pressure before and after PCI (OR 2.62, 95% CI 1.63–4.19), insulin treatment (OR 1.84, 95% CI 1.36–2.47), and the amount of contrast media (OR 1.30, 95% CI 1.16–1.46) [11]. Furthermore, it was reported that diabetes mellitus with CKD was a risk factor for CIN but that diabetes alone and CKD alone were not [12]. Furthermore, in a meta-analysis of 16 randomized controlled trials (RCTs) (total of 2727 patients) using iso-osmotic contrast media, iodixanol, or low-osmotic contrast agent, the prognostic factors for CIN were CKD, diabetes mellitus with CKD, and the use of low-osmotic contrast media [13].

In a systematic review on CIN incidence and the risk factors for CIN, the CIN incidence rate, defined as an increase in SCr ≥ 0.5 mg/dL or ≥ 25%, was 4.96% (95% CI 3.79–6.47). In terms of the risk factors, existing renal impairment (OR 1.73, 95% CI 1.06–2.82), diabetes mellitus (OR 1.87, 95% CI 1.55–2.26), malignant tumor (OR 1.79, 95% CI 1.03–3.11), aged over 65 years (OR 1.95, 95% CI 1.02–3.70), and use of non-steroidal anti-inflammatory drugs (NSAIDs) (OR 2.32, 95% CI 1.04–5.19) were related to CIN onset, whereas hypertension, anemia, and congestive heart failure were not related to CIN [14]. Moreover, in a systematic review on the CIN onset and the need for dialysis in patients receiving contrast CT, the CIN incidence rate was 6.4% (95% CI 5.0–8.1), but the need for dialysis was as low as 0.06% (95% CI 0.01–0.4). In that study, CKD (OR 2.26, 95% CI 1.66–3.07) and diabetes mellitus (OR 3.10, 95% CI 2.34–4.09) were risk factors for CIN [15].

As mentioned above, aging and diabetes mellitus are the main risk factors for CIN onset, though contradictory evidence has accumulated as to whether diabetes mellitus without CKD is a risk factor for CIN onset, and it remains unclear at this time. In addition, since there is no evidence of an increased risk of developing CIN based on age among aging patients, the degree of risk must be judged according to each individual’s condition.

### 3.4 **CQ3-4** Does the use of renin-angiotensin system (RAS) inhibitors increase the risk of developing CIN?

Answer:

There is no evidence that RAS inhibitors increase the risk of developing CIN.

**Level of Evidence: II/Grade of Recommendation: Not applicable**

**Rationale CQ3-4**

There is no evidence that the use of RAS inhibitors increases the risk of developing CIN. There is also no consensus as to whether RAS inhibitors increase the incidence of CIN [16, 17]. In recent years, 3 meta-analyses/systematic reviews were reported that examined the incidence of CIN in coronary angiography (CAG) (including arterioplasty). Zhou et al. reported [18] that the incidence of CIN was 7.9% under the continuous administration of ACE inhibitors (control: 8.2%, RR 0.95, and 95% CI 0.57–1.58). Jo et al. [19] reported that the use of RAS inhibitors was not a significant risk factor (OR 1.27, 95% CI 0.77–2.07, p = 0.351) in the meta-analysis. In the sub-analysis, classification of an RAS inhibitor continuation group and an initiation group showed that RAS inhibitor continuation was a significant risk factor for CIN as compared with RAS inhibitor discontinuation (OR 2.06, 95% CI 1.62–2.61), but that the initiation of RAS inhibitors was not a risk factor compared with placebo (OR 0.52, 95% CI 0.23–1.16, p = 0.108). Among the reports included in this meta-analysis, all reports showing the risk of CIN upon continuation of the RAS inhibitor were cohort studies, and the RCTs did not demonstrate a risk of developing CIN. These results are based on the sub-analysis, and further verification is necessary to draw definitive conclusions. In a meta-analysis of RCTs investigating CIN development [20], ACE inhibitors were not significant risk factors (OR 1.06, 95% CI 0.69–1.61, p = 0.8). In addition, no significant difference in CIN onset was observed between the continuation and discontinuation groups among patients receiving ACE inhibitor/angiotensin II receptor blocker therapy on CAG [21–23]. Considering the above reports, it is not clear whether RAS inhibitors increase the risk of developing CIN.

### 3.5 **CQ3-5** Does the continuation of diuretics increase the risk of developing CIN?

Answer:

It is not clear whether the continuation of oral diuretics increases the risk of developing CIN.

**Level of Evidence: II/Grade of Recommendation: Not applicable**

**CQ3-6 Does the prophylactic use of diuretics increase the risk of developing CIN?**

Answer:

Prophylactic use of diuretics is not recommended because it increases the risk of developing CIN.

**Level of Evidence: II/Grade of Recommendation: C2**

**Rationale CQ3-5**

A propensity score-matched cohort study (10,121 patients underwent contrast-enhanced CT and 10,121 patients underwent plain CT) showed that in patients with SCr > 1.6 mg/dL, AKI risk after CT was significantly high (OR 1.45, 95% CI 1.11–1.89, p = 0.007) and that the use of contrast media was not related to the onset of AKI after CT (p = 0.42) [24]. On the contrary, the use of diuretics was a significant risk factor for AKI after CT irrespective of the use of contrast media (OR 2.25, 95% CI 2.00–2.53, p < 0.001) [24].

Furthermore, the incidence of CIN was examined in 240 patients undergoing elective PCI who received furosemide or captopril and who were divided into 4 groups (60 patients with discontinuation of captopril, 60 patients with continuation of captopril, 60 patients with continuation of furosemide, and 60 patients with discontinuation of furosemide, at 36 h before PCI) [25]. There was no significant difference in the incidence of CIN in the furosemide or captopril discontinuation and continuation groups (3.3% in the captopril discontinuation group, 3.3% in the captopril continuation group, 3.3% in the furosemide discontinuation group, 1.6% in the furosemide continuation group). Thus, it is unclear whether the continuation of diuretics increases the risk of developing CIN following intravenous and intra-arterial administration of contrast media.

**Rationale CQ3-6**

It has been reported that treatment with loop diuretics to prevent CIN actually increased the incidence of CIN, even in patients without dehydration [26], In a study in which patients received hydration with 0.45% saline or 0.45% saline plus loop diuretics, the incidence of CIN was significantly higher in those receiving loop diuretics than in those receiving saline alone [27]. In recent observational studies of CAG including recent angioplasty, the use of furosemide in the perioperative period was an independent risk factor for CIN [28]. On the other hand, the incidence of CIN decreased significantly in patients receiving a combination of aggressive saline infusion and furosemide administration through devices that balanced high urine output and venous fluid infusion to maintain a urine output of 300 mL/h (see “Renal Guard therapy”).
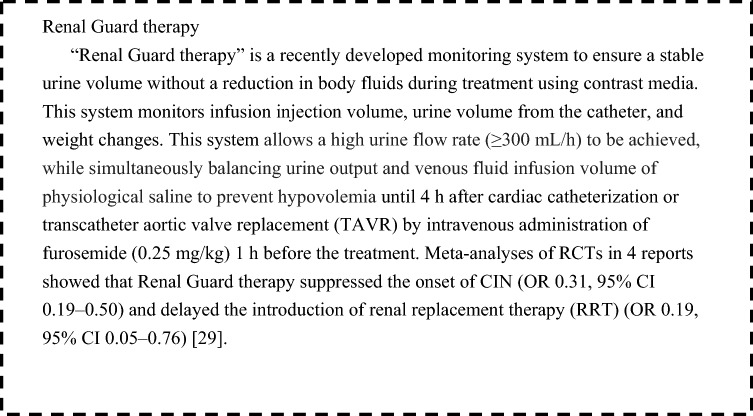


### 3.6 **CQ3-7** Does the use of NSAIDs increase the risk of developing CIN?

Answer:

We recommend against the use of NSAIDs owing to an increased risk of developing CIN.

**Level of Evidence: II/Grade of Recommendation: C2**

**Rationale CQ3-7**

The development of CIN is more frequently observed in patients taking NSAIDs [14, 30]. In a meta-analysis analyzing 18,790 patients who underwent intravenous contrast CT, 4.96% of patients developed CIN and the OR of NSAIDs use leading to CIN onset was 2.32 (95% CI 1.04–5.19) [14]. Discontinuation of NSAIDs 24 h before and after the use of contrast media is recommended [31, 32].

### 3.7 **CQ3-8** Does the use of iodinated contrast media increase the risk of lactic acidosis in patients receiving biguanide anti-hyperglycemic drugs?

Answer:

Biguanide anti-hyperglycemic drugs increase the risk of developing lactic acidosis when a transient decrease in kidney function occurs after the use of iodinated contrast media. When administering an iodinated contrast media, it is recommended that appropriate measures such as a temporarily withdrawal of biguanide anti-hyperglycemic drugs, except for emergency examinations, are considered in light of the risk of CIN.

**Level of Evidence: IV/Grade of Recommendation: C2**

**Rationale CQ3-8**

Lactic acidosis is one of the most serious adverse drug reactions to biguanide anti-hyperglycemic drugs. Although the incidence is very low, the prognosis of lactic acidosis is poor, and the mortality is high. Conditions that may lead to lactic acidosis include kidney disease (as biguanides are excreted unchanged through the kidneys, biguanide concentration in the blood may increase in patients with kidney dysfunction), liver disease (hepatic dysfunction decreases lactic acid metabolism in the liver), heart failure, myocardial infarction, and respiratory failure (hypoxemia may occur and accelerate anaerobic glycolysis, which increases the production of lactic acid). In Japan, biguanides are contraindicated for patients with a high risk of developing lactic acidosis. Currently, the risk of lactic acidosis due to biguanides is very low when these drugs are used according to the approved indications. However, when patients receiving biguanides develop AKI due to the use of iodinated contrast media, renal excretion of biguanides may decrease and lactic acidosis may develop. Cases of biguanide-associated lactic acidosis occurring after AKI due to the use of iodinated contrast media in patients with conditions known to increase the risk of lactic acidosis have been reported [33, 34]. Reviews of case series of CIN in patients receiving biguanides have been published [35–37]. However, among 372 patients with type 2 diabetes who underwent PCI, 23 (16%) of the metformin-taking patients (145 patients) developed CIN, but none developed lactic acidosis [38]. Guidelines published in Western countries [39–41] recommend that measures be taken for patients receiving biguanides who are going to receive iodinated contrast media. Although the recommended measures vary among guidelines, most guideline documents do not recommend the suspension of biguanides in patients with normal kidney function before the use of iodinated contrast media.

The second paragraph of the ‘‘Important Precautions’’ section of the package inserts for biguanides in Japan describes that ‘‘because patients receiving biguanides may develop lactic acidosis after the use of iodinated contrast media, treatment with biguanides should be suspended before contrast radiography (except for patients requiring emergency radiography)’’. Biguanides have become contraindicated in patients with moderate or greater renal dysfunction. Treatment with biguanides should not be resumed during the 48 h after the use of iodinated contrast media, and physicians should carefully observe patients when treatment with biguanides is resumed. Metformin is the most commonly used biguanide. The ‘‘Recommendations for Appropriate Use of Metformin’’ revised on May 12, 2016 by the committee on the appropriate use of biguanides (available in Japanese at the website of the Japan Diabetes Society: http://www.fa.kyorin.co.jp/jds/uploads/recommendation_metformin.pdf) describe that kidney dysfunction is common among patients with lactic acidosis associated with the use of biguanides and indicate that attention should be paid to the risk of an acute exacerbation of kidney dysfunction after the use of iodinated contrast media in patients receiving biguanides. Accordingly, the present guideline recommends that patients using biguanides discontinue the drugs prior to the use of iodinated contrast media, except for cases requiring emergency contrast radiography, and undergo other appropriate measures to prevent CIN.

### 3.8 **CQ3-9** Does the development of CIN worsen the vital prognosis of patients with CKD?

Answer:

The development of CIN may adversely affect the vital prognosis of patients with CKD, and the prognosis of CKD patients with CIN is poor. However, it is unclear whether CIN is a factor that defines or predicts the prognosis.

**Level of Evidence: IVa/Grade of Recommendation: Not applicable**

**Rationale CQ3-9**

Although it is believed that CIN is transient and kidney function recovers in most patients with CIN, many reports have indicated that the development of CIN affects vital prognosis [42–51]. In a prospective study of 78 patients with CKD who underwent CAG, the mortality at 5-year follow-up was significantly higher among the 10 patients who developed reversible AKI (90%) as compared with the 68 patients who didn't develop AKI (32%) [43]. In a retrospective case-matched cohort study of 809 patients who developed CIN after CT, CT angiography (CTA), angiography, contrast venography, or cardiac catheterization (53% of them received intravenous contrast media) and 2427 patients who did not develop CIN after contrast exposure, 1-year mortality was significantly higher in patients with CIN (31.8%) than in those without CIN (22.6%) [44]. In a study of the effects of CIN after the use of ioxaglate on the morbidity and mortality of 439 patients undergoing PCI, the cumulative 1-year mortality was significantly higher in the 161 patients with CIN (37.7%) than in the 278 patients without CIN (19.4%) [45]. In a study of 338 consecutive patients with acute coronary syndrome (ACS) undergoing emergency PCI, the in-hospital mortality was significantly higher in the 94 patients with CIN (9.6%) than in the 244 patients without CIN (3.3%) [46].

Although it is believed that the incidence of CIN is lower in patients who receive contrast media intravenously than in those who receive it intra-arterially, few reports have described the incidence of CIN and its effect on vital prognosis in patients receiving intravenous contrast media, and no consensus has been reached regarding any difference in CIN incidence according to the route of administration [52, 53].

In a study of 421 patients with an eGFR < 60 mL/min/1.73 m^2^ who underwent contrast-enhanced CT with intravenous iodinated contrast media, no significant correlation was observed between the incidence of CIN and the 30-day mortality [54]. In a 1-year retrospective review of 1184 trauma patients who received intravenous contrast media, the in-hospital mortality was significantly higher in the 78 patients with CIN (9.0%) than in those without CIN (3.2%), but a logistic regression analysis revealed no significant correlation between in-hospital mortality and CIN [55]. In a study of 139 patients undergoing contrast-enhanced CT in an intensive care unit (ICU) setting, the ICU mortality and in-hospital mortality in the 16 patients with CIN (31% and 50%, respectively) tended to be higher than those in the 123 patients without CIN (13% and 26%, respectively), but no statistically significant differences in these variables were observed (p = 0.068 and p = 0.074, respectively) [56]. Recently, a large-scale retrospective observation study (6902 patients) compared the risk of CIN development, dialysis within 30 days, and mortality between a contrast-enhanced CT group and a plain CT group. Patients were subdivided into CKD stage G3 (2440 patients) and CKD stage G4-5 (838 patients), and propensity score (1:1) matching was conducted to adjust for the background factors. As a result, there was no significant difference in at least the short-term vital prognosis between the contrast-enhanced CT group and plain CT group in any subgroup [57]. It is not clear whether the onset of CIN by intravenous contrast agent worsens the vital prognosis.

While many reports have described a relationship between CIN and vital prognosis, as mentioned previously, it remains unclear whether CIN defines prognosis (i.e., the occurrence of CIN worsens the vital prognosis) or predicts prognosis (i.e., CIN occurs in patients with poor vital prognoses).

### 3.9 **CQ3-10** Does the use of contrast media increase the risk of a decline in residual kidney function in patients undergoing peritoneal dialysis?

Answer:

Although the use of contrast media may be a risk factor for a decline in residual kidney function in patients undergoing peritoneal dialysis, it has been reported that radiography using only 100 mL of contrast media does not affect residual kidney function when urine output is adequately maintained.

**Level of Evidence: IVa/Grade of Recommendation: Not applicable**

**Rationale CQ3-10**

Only a few reports have been published regarding the effect of iodinated contrast media in patients receiving peritoneal dialysis who have some residual kidney function. It has been reported that the use of approximately 100 mL of contrast media did not decrease residual kidney function in patients undergoing peritoneal dialysis with a creatinine clearance (CCr) of 4.4–7.0 mL/min/1.73 m^2^ compared with the control group [58, 59]. Urine volume had a range of 1300–1800 mL/day in many of the patients enrolled in these studies. It is unclear why the use of contrast media did not deteriorate kidney function in these patients with severe kidney dysfunction (CKD G5D). Further studies should be conducted to clarify the exact reasons, e.g., maintenance of urine volume, slow removal of contrast media through peritoneal dialysis, or alkalemia frequently observed in patients undergoing peritoneal dialysis. Little evidence has been obtained regarding the effect of contrast media in patients with a urine volume of lower than 1000 mL/day. Further studies should be conducted to investigate the effects of contrast media in patients with a CCr lower than 4.0 mL/min/1.73 m^2^ or in those with decreased residual kidney function and to specify the tolerable volume of contrast media for patients with varying degrees of residual kidney function.

### 3.10 **CQ3-11** Are risk scores useful as predictors of CIN development?

Answer:

Although it has been reported that several risk scores are available as predictors of the development of CIN after CAG or PCI, their usefulness has not been thoroughly investigated. It is inappropriate to recommend the use of such risk scores at the present time.

**Level of Evidence: IVa/Grade of Recommendation: Not applicable**

**Rationale CQ3-11**

Many investigators have reported on risk scoring for CIN onset. The following risk scoring system was reported to be useful for assessing the risk of CIN onset after PCI or the need for dialysis: (hypotension, 5 points; intra-aortic balloon pump (IABP), 5 points; congestive heart failure, 5 points; aged ≥ 75 years, 4 points; anemia, 3 points; diabetes mellitus, 3 points; 1 point per 100 mL of contrast media; for renal function: SCr ≥ 1.5 mg/dL, 4 points or 2 points for eGFR (mL/min/1.73 m^2^) 40–60, 4 points for eGFR 20–40, 6 points for eGFR < 20) [60, 61]. The risks of CIN onset and dialysis initiation based on this risk scoring were reported to be 7.5% and 0.04% at 5 points or less, 14.0% and 0.12% at 6–10 points, 26.1% and 1.09% at 11–15 points, and 57.3% and 12.6% at ≥ 16 points, respectively (Table 2) [60]. Although this report was validated at different facilities and showed high external validity, the number of samples was not statistically sufficient. In addition, because the amount of contrast media used is included in risk scores, the risk assessment is not viable for used before CAG or PCI.

**Table 2:** CIN risk scores

Risk factor	Integer score
Hypotension	5
IABP use	5
CHF	5
Age > 75 years	4
Anemia	3
Diabetes	3
Contrast media volume	1 for 100 mL
SCr level > 1.5 mg/dL or eGFR (mL/min/1.73 m^2^)	4 2 for 40 to 60 4 for 20 to < 40 6 for < 20

Risk score	Risk for CIN (%)	Risk for dialysis (%)
Total score		
0–5	7.5	0.04
6–11	14.0	0.12
11–15	26.1	1.09
≥ 16	57.3	12.6

*IABP* intra-aortic balloon pumping, *CHF* congestive heart failure, *SCr* serum creatinine, *eGFR* estimated glomerular filtration rate, *CIN* contrast induced nephropathy

In recent systematic reviews, Sliver et al. [62] and Allen et al. [63] compared 12 and 74 risk scoring systems, respectively, and revealed that some risk scores can be expected to be useful. The analysis by Allen et al. showed that the usefulness of the risk score did not decrease when the contrast media amount was not included [63] and that a risk assessment can be preoperatively conducted to facilitate CIN prediction and CIN prevention by intravenous hydration.

Brown et al. examined preoperatively calculated risk scores without assessing the amount of contrast media using 110,000 cases in a CAG/PCI cohort. External validity of this risk score has been confirmed in different cohorts (20,800 patients), and the C-statistic for AKI onset was 0.74 (95% CI 0.74–0.75), suggesting that this score is useful [64]. In addition, Tsai et al. divided 90,500 PCI patients into a derivation cohort and a validation cohort using a cohort from the National Cardiovascular Data Registry Cath-PCI (NCDR Cath/PCI) and create d a new risk score (Table 3) [65]. As in the report from Brown et al., this risk score does not include the amount of contrast media as a risk factor and it can be calculated preoperatively. The C-statistic for CIN onset was 0.71 (95% CI 0.71–0.72), which indicates a useful risk score. In addition, this risk score was examined in 11,000 PCI patients in multicenter using the JCD-KiCS (Japan Cardiovascular Studied) registry [66]. The C-statistic for the onset of CIN was 0.76, and its usefulness was also supported in the verification of the calibration plot. However, since only PCI patients were examined in these studies, it has not been verified whether this risk score can be applied to CAG examination.

**Table 3:** CIN risk scores

	Score	
	AKI onset	Dialysis initiation
Age (years)		
< 50	0	
50–59	2	
60–69	4	
70–79	6	
80–89	8	
≥ 90	10	
Prior 2 weeks HF	2	2
eGFR < 30 mL/min/1.73 m^2^	5	5
eGFR < 30–45 mL/min/1.73 m^2^	3	3
eGFR < 45–60 mL/min/1.73 m^2^	1	1
Diabetes	7	1
Prior HF	4	
Prior CVD	4	
NSTEMI/UA	6	1
STEMI	15	2
Prior card shock	16	
Prior card arrest	8	3
Anemia (Hb < 10 g/dL)	10	
IABP	11	

AKI score	Risk (%)	Dialysis score	Risk (%)
Risk for CIN and dialysis as risk scores			
0–4	1.9	0	0.03
5	2.6	1	0.05
10	3.6	2	0.09
15	4.9	3	0.15
20	6.7	4	0.27
25	9.2	5	0.48
30	12.4	6	0.84
35	16.5	7	1.5
40	21.7	8	2.6
45	27.9	9	4.4
50	35.1	10	7.6
55	43.0	11	12.6
> 60	51.4	12	20.3
		13	31.0

*NSTEMI/UA* non-ST elevation myocardial infarction/unstable angina, *STEMI* ST elevation myocardial infarction, *IABP* intra-aortic balloon pumping, *eGFR* estimated glomerular filtration rate, *CIN* contrast induced nephropathy, *AKI* acute kidney injury, *HF* heart failure, *CVD* cardiovascular disease

In order for the risk score to become established in the clinical setting in the future, the clinical usefulness (external validity) of the score should be validated at multiple centers, and a tool for easy calculation of the risk score should also be disseminated [62].

### 3.11 **CQ3-12** Does a solitary kidney increase the risk of developing CIN?

Answer:

The evidence that a solitary kidney increases the risk of developing CIN compared to having both kidneys is not clear.

**Level of Evidence: IVa/Grade of Recommendation: B**

**(Minds 2017) Quality of evidence: C Strength of recommendation: not applicable**

**Rationale CQ3-12**

Donor or recipient kidney transplantation patients and patients with renal excision due to malignant tumors, etc., have been considered to be at higher risk of CIN since the renal function of patients with solitary kidney is lower than that of patients with bilateral kidneys. However, to clarify whether the solitary kidney itself increases the risk of developing CIN, it is necessary to consider the various risk factors for CIN onset, including basal renal function.

Recently, Cheungpasitporn et al. reported a meta-analysis of CIN onset in renal transplant recipients [67]. Six studies of 431 kidney transplant recipients were analyzed, excluding one study that used contrast media in the early stage (within 1–2 months after transplantation). The CIN incidence rate was 9.6% (coronary angiography, 16.1%; angiography, 10.1%; contrast-enhanced CT, 6.1%). However, this study was only a descriptive study on the incidence of CIN in transplant recipients, and there was no comparison to the incidence in patients with bilateral kidneys.

McDonald et al. retrospectively examined 6175 patients who received contrast-enhanced CT in a single facility [68]. Propensity score (1:3) matching was performed to adjust for basal renal function and underlying diseases, and 247 patients with solitary kidney and 691 patients with bilateral kidneys were compared. Enhanced CT was performed 30 days after nephrectomy (76% due to malignant tumor; 5% in transplant recipients; 19% in others). As a result, there was no significant difference in the CIN onset between patients with a solitary kidney (4.1%) and bilateral kidneys (4.2%) (OR 0.96, 95% CI 0.41–2.07). However, in that study, the proportion of patients with an eGFR > 60 mL/min/1.73 m^2^, eGFR 30–59 mL/min/1.73 m^2^, and eGFR < 30 mL/min/1.73 m^2^ was 47, 52, and 0.4%, respectively. Therefore, the risk in patients with an eGFR < 30 mL/min/1.73 m^2^ is unknown due to the current lack of evidence. Whether solitary kidney is a risk factor for CIN at this time remains unclear, and further study is warranted.

## 4 Type and volume of contrast media

### 4.1 **CQ4-1** Does the risk of developing CIN differ between iso- and low-osmolar contrast media?

Answer:

The risk of developing CIN does not differ between iso- and low-osmolar contrast media.

**Level of Evidence: II/Grade of Recommendation: Not applicable**

### 4.2 **CQ4-2** Does the risk of developing CIN differ among the different low-osmolar contrast media?

Answer:

Although there has been no definitive conclusion as to whether the risk of developing CIN differs among the different types of low-osmolar contrast media, there has been no significant difference in the incidence of CIN among them.

**Level of Evidence: II/Grade of Recommendation: Not applicable**

**Rationale CQ4-1 to 4-2**

In a randomized, double-blind, prospective, multicenter study comparing the nephrotoxic effects of an iso-osmolar contrast medium (iodixanol) with those of a low-osmolar contrast medium (iohexol) in 129 patients with diabetes with an SCr level of 1.5–3.5 mg/dL who underwent CAG or aorto-femoral angiography, 2 of the 64 patients in the iodixanol group (3.1%) showed increased SCr levels (≥ 0.5 mg/dL) as compared with 17 of the 65 patients in the iohexol group (26.2%) (p = 0.002), suggesting that CIN may be less likely to develop when an iso-osmolar contrast medium is used rather than a low-osmolar contrast medium [69]. However, there were no differences in the incidence of CIN onset between iso-osmolar contrast media and low osmolar contrast media in 25 randomized controlled prospective trials and 2 meta-analyses, and the use of iso-osmolar contrast media was not found to reduce the CIN risk (OR 0.87, 95% CI 0.73–1.04) [69–95].

Although there is no clear evidence as to the CIN risk among the various low-osmolar contrast media, many current reports have indicated no difference in the CIN risk [71, 96, 97].

### 4.3 **CQ4-3** Is the risk of developing CIN higher in patients receiving contrast media via intra-arterial administration than in those receiving contrast media via intravenous administration?

Answer:

Although there is no evidence demonstrating that intra-arterial administration of contrast media is an independent risk factor for the development of CIN, the incidence of CIN tends to be higher in patients receiving contrast media intra-arterially than in those receiving them intravenously. Since this tendency may be based on differences in the underlying diseases of the patients (including diabetes and chronic kidney diseases), it is necessary to consider the patient’s underlying disease, etc., especially when contrast media are intra-arterially administered.

**Level of Evidence: IVa/Grade of Recommendation: Not applicable**

**Rationale CQ4-3**

The majority of studies on CIN have been conducted in patients receiving contrast media intra-arterially, and only a few studies have investigated a possible difference in the incidence of CIN according to the route of administration. In a review of 7 prospective observational studies, the overall incidence of CIN was lower in CKD patients who received intravenous low- or iso-osmolar contrast media, suggesting that intravenous administration may pose a smaller risk of CIN as compared with that posed by intra-arterial administration [50].

In recent years, it has been reported that there are few direct comparisons of the risk of developing CIN according to differences in the administration route [98–103]. A retrospective examination [103] showed that there was no difference in the incidence of CIN among the 1969 patients who received both intra-arterial administration and intravenous administration and that patient background factors such as diabetes and chronic heart failure may be implicated in the onset of CIN.
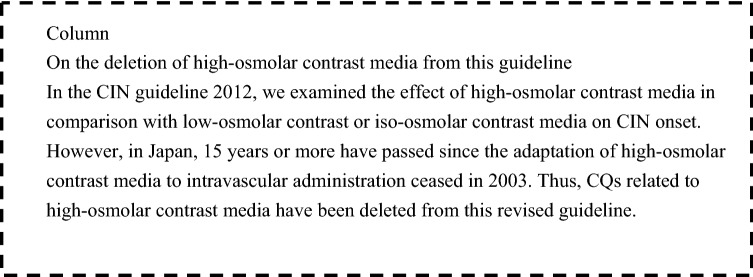


## 5. Intra-arterial contrast media administration

### 5.1 **CQ5-1** Does the risk of developing CIN after CAG increase in CKD patients?

Answer:

1. It is highly likely that the risk of developing CIN after CAG increases in CKD patients (eGFR < 60 mL/min/1.73 m^2^), as the risk increases as kidney function decreases.
2. We recommend that physicians explain CIN to patients with an eGFR < 60 mL/min/1.73 m^2^ who will undergo CAG and that they emphasize the need for appropriate preventive measures such as fluid hydration before and after CAG.

**Level of Evidence: I/Grade of Recommendation: A**

**Rationale CQ5-1**

Recently, CAG and catheter-based revascularization have become common procedures, and the use of contrast media has increased substantially. It has been reported that the risk of CIN increases in CKD patients as kidney function (GFR) decreases [10]. In 2001, Shiraki et al. reported that 61 of 1920 patients (3.2%) who underwent CAG developed CIN, 1 of whom (0.05%) required hemodialysis. In another study, Fujisaki et al. reported that CIN developed in 12 of 267 patients (4.5%) who underwent CAG and that hemodialysis was required in 2 patients (0.7%). In a report from the Mayo Clinic in 2002, CIN developed in 254 of 7586 patients (3.3%) who underwent CAG, and 20 patients (7.9%) required hemodialysis [104]. Mortality at 1 and 5 years in patients with CIN was 12.1% and 44.6%, respectively, which were both significantly higher than those in patients without CIN (3.7% and 14.5%, respectively). In a study reported in 2009, Abe et al. [105] reported that the incidence of CIN within 5 days after CAG was 4.0% in 1157 consecutive patients who underwent CAG and that the risk factors for CIN included a baseline SCr (≥ 1.2 mg/dL) and the use of a large volume of contrast media (≥ 200 mL).

**Fig. 1:**
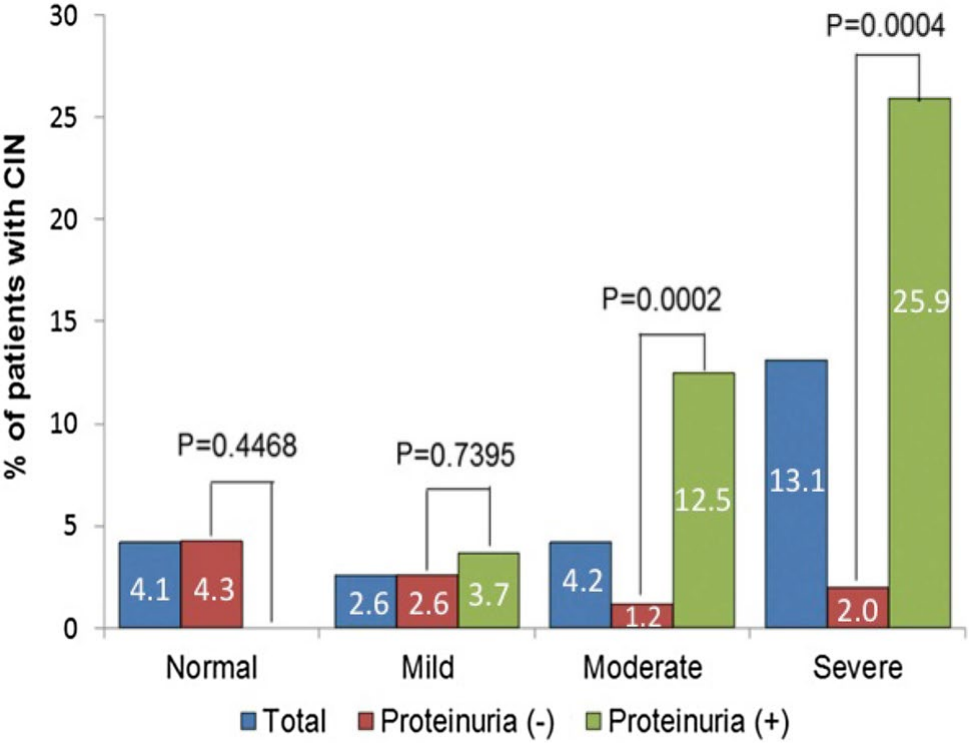
Incidence of CIN according to renal function. The incident rate increased as eGFR decreased, and proteinuria was an independent risk factor for CIN

Recently, the CINC-J study, a prospective multicenter study of Japan, examined the incidence of CIN in 907 patients who underwent cardiac catheterization and demonstrated that the incidence of CIN was 4.1% in patients with an eGFR ≥ 60 mL/min/1.73 m^2^, 2.6% in those with 45 ≤ eGFR < 60 mL/min/1.73 m^2^, 4.2% in those with 30 ≤ eGFR < 45 mL/min/1.73 m^2^, 13.1% in those with an eGFR < 30 mL/min/1.73 m^2^. Although CIN developed even in patients with normal renal function, the incident rate increased as eGFR decreased, and proteinuria was an independent risk factor for CIN [106] (Fig. 1).

CIN developed mainly in high-risk patients such as those with diabetes, anemia, dehydration, or underlying kidney diseases and/or those who were elderly or were receiving nephrotoxic agents [61]. It is recommended that patients with CKD should receive appropriate preventive treatment such as fluid hydration therapy and be closely monitored for kidney function after CAG.

### 5.2 **CQ5-2** Does the use of a smaller volume of contrast media decrease the risk of developing CIN?

Answer:

Since the use of a smaller volume of contrast media has been found to decrease the risk of developing CIN in patients undergoing CAG, we recommend that contrast media should be administered at the minimum necessary volume.

**Level of Evidence: II/Grade of Recommendation: A**

**Rationale CQ5-2**

**Fig. 2:**
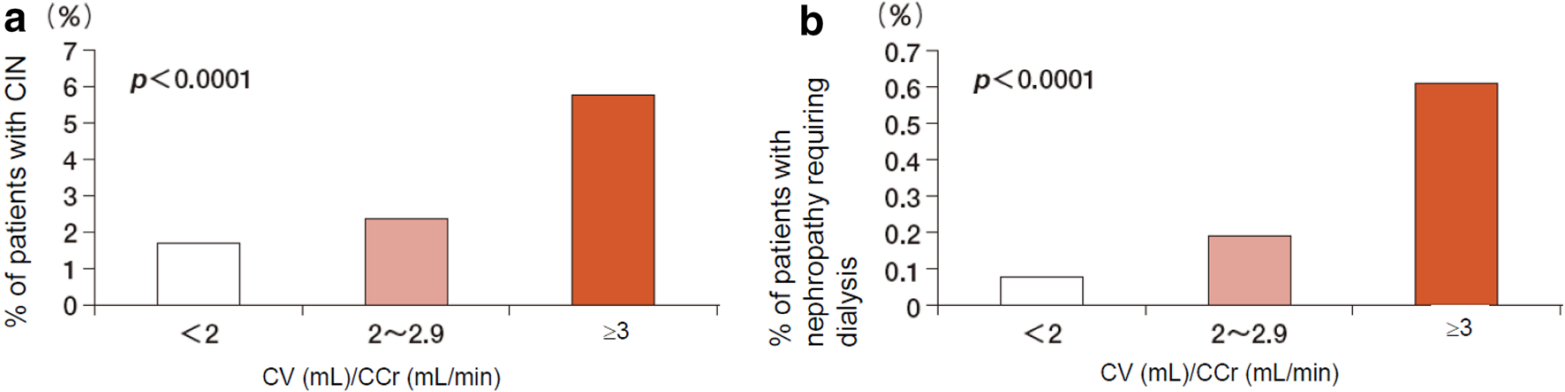
Incidences of CIN and nephropathy requiring dialysis (NRD). Incidences of CIN and NRD increased in patients with higher CV/CCr values (renal function) and were especially high in patients with a CV/CCr of ≥ 3. *CV* contrast volume, *CCr* calculated creatinine clearance. Adapted from a study [110]

Since the risk of developing CIN increases as the dose of contrast media increases, unnecessary use of contrast media should be avoided in all patients. Although the volume of contrast media used in CAG ranges from 50 to 100 mL in many patients, it is recommended that contrast media used for patients with CKD should be limited to the minimum necessary volume. In a study of 10,065 patients undergoing PCI, Brown et al. [107] reported that the incidence of AKI was significantly higher in patients who received doses of contrast media above the minimum necessary volume than in those who received doses below it. Nyman et al. [108] suggested that the contrast media dose-to-eGFR ratio (gram-iodine/eGFR) should be kept under 1.0 (see **CQ6-3**), and Laskey et al. [109] recommended that the ratio of the volume of contrast media to the CCr should be limited to < 3.7. Some reports have advocated lower ratios of the contrast media volume to the CCr. In a study of 58,957 patients undergoing PCI, the risk of CIN and nephropathy requiring dialysis (NRD) approached significance when the contrast dose-to-CCr ratio exceeded 2.0 and was dramatically elevated in patients exceeding a contrast dose-to-CCr ratio of 3.0 (Fig. 2) [110]. On the basis of these findings, it is recommended that the volume of contrast media used during CAG or PCI be limited to the minimum necessary volume in patients with CKD (see **CQ6-3**) [10].

### 5.3 **CQ5-3** Does repeated CAG at short intervals increase the risk of developing CIN?

Answer:

Since repeated CAG at short intervals may increase the risk of developing CIN, we do not recommend repeating CAG within a short time interval (24–48 h) in CKD patients with an eGFR < 60 mL/min/1.73 m^2^).

**Level of Evidence: VI/Grade of Recommendation: C2**

**Rationale CQ5-3**

Since it has been reported that repeated CAG within 24–48 h may increase the risk of developing CIN, patients with CKD should not undergo repeated CAG in a short time interval (24–48 h; see **CQ6-5**). There have been no studies investigating the effect of repeated CAG within 1 year on the risk of developing CIN.

### 5.4 **CQ5-4** Does the risk of developing CIN after PCI increase in CKD patients?

Answer:

In CKD patients with an eGFR < 60 mL/min/1.73 m^2^, the risk of CIN onset increases after PCI. However, there is no evidence demonstrating that PCI itself worsens the prognosis of CKD patients.

**Level of Evidence: I/Grade of Recommendation: A**

**Rationale CQ5-4**

In a study of 439 patients with baseline SCr levels ≥ 1.8 mg/dL who underwent PCI, Gruberg et al. [45] reported that 161 patients (36.7%) experienced CIN and 31 (7.1%) required hemodialysis. In-hospital mortality was 14% for patients with further kidney function deterioration after PCI. In a study of 208 consecutive patients with acute myocardial infarction undergoing primary PCI, Marenzi et al. [48] reported that CIN developed in 40 patients (19.2%). Of the 160 patients with a baseline eGFR < 60 mL/min/1.73 m^2^, CIN developed in 21 patients (13.1%), whereas it developed in 19 (39.6%) of the patients with an eGFR < 60 mL/min/1.73 m^2^. The risk factors for CIN included age ≥ 75 years, use of ≥ 300 mL of contrast media, 6 h of time-to reperfusion, presence of anterior myocardial infarction, and use of an IABP, and CKD was not a significant risk factor for CIN. In 2005, Dangas et al. [42] investigated 7230 patients undergoing PCI and reported that CIN developed in 381 of 1980 patients (19.2%) with a baseline GFR < 60 mL/min/1.73 m^2^ and 688 of 5250 patients (13.1%) with a baseline GFR ≥ 60 mL/min/1.73 m^2^.

Recently, large-scale cohort studies have been reported. The Blue Cross Blue Shield of Michigan Cardiovascular Consortium study reported that CIN occurred in 1234 (2.57%) out of 48,001 PCI patients and that dialysis was initiated in 169 patients (0.35%) [111]. In addition, the National Cardiovascular Data Registry Cath-PCI registry study reported that CIN occurred in 69,658 patients (7.1%) and 3005 (0.3%) patients started dialysis among 985,737 patients who underwent PCI [112]. In terms of renal function, the incidence rates of CIN were 5.2% in patients with an eGFR ≥ 60 mL/min/1.73 m^2^, 8.0% in those with 45 ≤ eGFR < 60 mL/min/1.73 m^2^, 12.9% in those with 30 ≤ eGFR < 45 mL/min/1.73 m^2^, and 26.6% in those with an eGFR < 30 mL/min/1.73 m^2^. The CREDO-Kyoto registry in Japan reported a 5% incidence rate of CIN in 4371 patients who underwent PCI and that CIN occurred significantly more frequently in CKD patients (11%) than in non-CKD patients (2%) [113]. In addition, in a systematic review analyzing a risk prediction model for CIN, 3062 (4.2%) of 72,214 subjects developed CIN [114] and the risk factors for CIN were identified as CKD, age, diabetes, heart failure, low heart function, hypotension, and shock.

In 2010, Chong et al. [115] investigated a cohort of 8798 patients who underwent PCI and reported that the incidence of CIN was high in patients with a baseline eGFR of < 30 mL/min/1.73 m^2^ as well as in patients who underwent emergency or elective PCI. Among ST elevation myocardial infarction (STEMI) patients undergoing primary PCI, unstable angina pectoris/non-STEMI patients undergoing early PCI, and patients without myocardial infarction undergoing elective PCI, the incidence of CIN was 8.2, 9.2, and 4.3% in those with an eGFR ≥ 60 mL/min/1.73 m^2^ (p < 0.0005), 19.1, 4.5, and 2.4% in those with an eGFR 30–59 mL/min/1.73 m^2^ (p < 0.0005), and 34.4, 40.0, and 28.9% in those with an eGFR < 30 mL/min/1.73 m^2^ (p = 0.510), respectively. The incidence of CIN was significantly higher in emergency PCI for unstable angina pectoris and STEMI than in standby PCI for stable angina pectoris. In addition, the incidence in patients with an eGFR < 30 mL/min/1.73 m^2^ was high in all groups regardless of emergency PCI [115]. According to a Japanese report from Abe et al. in 2014, the incidence of CIN in patients with STEMI (16.1%), unstable angina pectoris (UAP)/non-STEMI (10.7%) was significantly more frequently than in patients with stable angina pectoris (SAP) (4.24%) [116]. In a multivariate analysis, emergency PCI, left ventricular ejection fraction < 40%, and anemia were independent risk factors for CIN. In a 2015 study investigating CIN and cardiovascular event occurrence in 9512 patients who underwent PCI for ACS, the incidence of CIN was 12.7% and the predictive factors for CIN onset were CKD, diabetes, contrast volume, age, left ventricular function, anemia, and others [117].

These findings indicate that the incidence of CIN and in-hospital mortality may be higher in patients undergoing emergency PCI for the treatment of acute myocardial infarction than in patients undergoing elective PCI for the treatment of stable angina, because the former patients have cardiac failure and unstable hemodynamics due to myocardial infarction and require a larger volume of contrast media (see **CQ6-5**). There is no evidence indicating that PCI itself worsens the prognosis of CKD. It is recommended that patients with coronary artery disease with indications for CAG and PCI understand the risk of post-procedural deterioration of kidney function, receive guidance as to appropriate preventive measures such as fluid therapy, and be exposed to the minimum necessary volume of contrast media [118].

### 5.5 **CQ5-5** How can CIN be differentiated from kidney injury due to cholesterol embolism?

Answer:

CIN may be differentiated from kidney injury due to cholesterol embolism on the basis of clinical and laboratory findings, although in some cases differentiation is difficult.

**Level of Evidence: IVb/Grade of Recommendation: Not applicable**

**Rationale CQ5-5**

Approximately 80% of the cases of cholesterol embolism are due to iatrogenicity, such as cardiovascular surgery and intravascular catheter examination [119]. The incidence of cholesterol embolism after catheterization was reported to be 1.4%, with a 0.9% frequency of renal dysfunction [120]. The clinical course of kidney injury also varies from the acute type that develops within 1 week, the subacute type that progresses over several weeks to several months, and the chronic type that progresses with a slow course [121]. Although reports have demonstrated that the prognosis is poor and the 1-year mortality rate is about 60–80%, improvements by up to 13% with multidisciplinary therapy were also reported [121]. About 30–60% of patients required dialysis when renal dysfunction occurred, and dialysis could be withdrawn in 20–40% of those patients [121]. Therefore, it is necessary to distinguish between CIN and cholesterol embolism in the context of renal dysfunction that develops after catheter examination to determine the appropriate treatment.

Kidney injury due to cholesterol embolism has the following key features that help to differentiate it from CIN:

1. Prolonged and progressive kidney dysfunction that develops several days or weeks after catheterization.
2. AKI that is often irreversible and sometimes follows a progressive course.
3. Multiple organ failure that may develop in addition to AKI.
4. Systemic symptoms of embolism such as livedo reticularis of the legs, cyanosis, and blue toes may develop.
5. Vasculitis-like symptoms such as fever, arthralgia, general malaise, eosinophilia, increased CRP, decreased serum complement, and elevated sedimentation rate may develop.
6. A diagnosis must be confirmed by pathological examination including skin and kidney biopsies.

Recently, it was reported that CAG and PCI through the transfemoral arterial approach had a higher incidence of AKI compared with the transradial arterial approach [122–124]. Since the catheter passes through the abdominal aorta and the descending aorta in the transfemoral arterial approach, it is possible that plaque at the aorta is scattered by the stimulation of the catheter, which leads to renal injury due to cholesterol embolism. Therefore, attention should be paid to the development of cholesterol embolism in cases where there is arteriosclerotic disease, and approaches from upper limbs should be considered in patients with aortic plaque.

### 5.6 **CQ5-6** Does the onset of CIN increase cardiovascular events?

Answer:

The incidence of cardiovascular events is high in patients who develop CIN.

**Level of Evidence: IVb/Grade of Recommendation: Not applicable**

**Rationale CQ5-6**

Several reports have investigated the association between CIN onset and cardiovascular events, and most have shown that CIN onset was associated with the subsequent occurrence of cardiovascular events [28, 117, 125–131].

James et al. conducted a meta-analysis and systematic review on the relationship between CIN and subsequent clinical outcome in patients undergoing CAG and demonstrated that CIN onset was associated with all-cause death and cardiovascular events [125].

In a study investigating the association between CIN and cardiovascular events (all-cause death, myocardial infarction, revascularization, stent thrombosis) in 9512 patients who underwent PCI for ACS, CIN occurred in 12.7% of patients and the incidence of cardiovascular events was significantly higher in CIN-onset patients than in the patients who did not develop CIN 1 year after PCI (22.0 vs. 15.4%, p < 0.0001). In addition, multivariate analysis showed that CIN was an independent predictor of cardiovascular events after adjusting for age, sex, diabetes, and the presence or absence of CKD [117].

A large-scale observational study investigating the association between AKI after PCI and cardiovascular events (all-cause death, myocardial infarction, hemorrhage requiring hospitalization within 1 year after discharge) was reported in 453,475 patients who underwent PCI. The frequency of AKI was 8.8%, and the severity of AKI was 7.5% in stage 1 and 1.2% in stage 2 or 3 by AKIN classification. The incidence of cardiovascular events was 11.1, 24.0, and 34.1% (p < 0.0001) 1 year after PCI in patients who did not develop AKI, those with AKIN stage 1, and patients with AKIN stage 2 or 3, respectively. With increasing severity, the incidence of cardiovascular events also increased. Furthermore, multivariate analysis of factors influencing cardiovascular events showed that AKI was an independent risk factor for cardiovascular events [126].

In addition, it is known that SCr elevation in CIN development is transient and renal dysfunction usually recovers [32]. A study investigating 1490 patients with an eGFR < 60 mL/min/1.73 m^2^ who received CAG showed that the incidence of all-cause death, dialysis, stroke, and myocardial infarction-combined endpoints within 5 years was significantly higher in 136 CIN patients who recovered from a transient rise in SCr and in 31 CIN patients who did not recover than in 1310 patients who did not develop CIN. A multivariate analysis adjusting for age, renal function, and cardiac function demonstrated that CIN onset were independent prognostic factors regardless of the recovery [127].

A nationwide Japanese cohort study, CINC-J, investigated the incidence of CIN and cardiovascular events (all-cause death, myocardial infarction, stroke, heart failure) in 853 cases in which CAG was performed in 27 facilities. The incidence of CIN was 5.2%, and the incidence of cardiovascular events (average observation period: 477 days) was significantly higher in patients who developed CIN than those who did not develop CIN (18 vs. 7.7%, p = 0.0451). In addition, it was reported that the combination of CIN and anemia was an independent predictor of cardiovascular events [128]. The CREDO-Kyoto registry, a cohort study of Japanese patients undergoing PCI/CABG, also examined the relationship between CIN and long-term prognosis and showed that the incidence of CIN in 4371 patients was 5% and that CIN was an independent predictor of all-cause mortality [105].

Thus, the incidence of cardiovascular events increases with the onset of CIN, though it remains unclear what kind of biological mechanisms affect the occurrence of cardiovascular events in CIN. Age, diabetes, decreased renal function, anemia, heart failure, etc., are all risk factors for cardiovascular events as well as for CIN. It is not clear whether patients who are likely to experience cardiovascular events have certain background factors that lead to CIN or whether CIN itself causes the increased likelihood of cardiovascular events.

### 5.7 **CQ5-7** Does the risk of developing CIN/AKI increase with TAVR in CKD patients?

Answer:

Few reports have shown that the risk of developing CIN/AKI increases with TAVR in CKD patients, though the risk of AKI does increase. CKD patients with an eGFR < 60 mL/min/1.73 m^2^ are more likely to have an increased risk of developing AKI by TAVR.

**Level of Evidence: IVb/Grade of Recommendation: Not applicable**

**Rationale CQ5-7**

Meta-analyses have shown that CKD patients treated with TAVR have a poor prognosis after treatment compared to those without CKD [131–133]. However, reports on the onset of CIN have been extremely limited. The investigations suggest that the rates of AKI onset are not very high, probably due to the short history of TAVR. In the AKI/CIN studies on TAVR, the evaluation of the deterioration of renal function varied; however, this guideline committee referred to the studies that classified CKD according to the eGFR.

One meta-analysis [131] showed that the risk of AKI was significantly higher in 2212 CKD patients (eGFR < 60 mL/min/1.73 m^2^) than in 2522 patients with an eGFR ≥ 60 mL/min/1.73 m^2^ (HR 1.42, 95% CI 1.20–1.68) by analyzing the AKI onset in TAVR in 6 studies [134–139]. In addition, they showed that the risk of AKI was significantly higher in 2512 CKD patients (eGFR < 60 mL/min/1.73 m^2^) than in 1874 patients with an eGFR ≥ 60 mL/min/1.73 m^2^ (HR 1.60, 95% CI 1.08–2.36) by analyzing the onset of severe AKI (stage 2 or 3) in TAVR in 7 studies [134–138, 140]. The report from the FRANCE2 registry analyzing 2929 patients showed that with the progression of the CKD stage (G 1-2, G 3 a, G 3 b, G 4, G 5) before TAVR, the risk of AKI became higher [141]. Furthermore, it has been reported that prognosis was poor in patients who developed AKI after TAVR [142, 143], suggesting that the prevention of AKI is important.

However, there are reports that TAVR is less likely to cause AKI than surgical aortic valve replacement surgery for aortic valve stenosis [144]. Originally, TAVR was performed for severe aortic valve stenosis patients whose hemodynamics change easily when anesthesia is introduced and/or after. In TAVR in particular, the following factors aside from the contrast medium may strongly influence the onset of AKI: (1) the use of equipment with a large diameter and poor mobility; (2) the insertion of large-diameter devices such as a 14-18 Fr sheath via the femoral artery approach, which may affect the TAVR patients with calcification of large vessels, wall plaques, thrombosis, etc.; and (3) bleeding due to open chest surgery in cases of the transapical approach. It is necessary to take these factors into consideration when interpreting the data. In addition to the TAVR procedure itself, effective management should consider that contrast-enhanced CT is necessary as a preoperative evaluation and most of the target patients are elderly.

## 6 Intravenous contrast media administration

### 6 1 **CQ6-1** Does the risk of developing CIN increase in CKD patients after contrast-enhanced CT?

Answer:

1. It is unlikely that the risk of developing CIN increases in CKD patients (eGFR ≥ 30 mL/min/1.73 m^2^) after contrast-enhanced CT. However, even if the eGFR is ≥ 30 mL/min/1.73 m^2^, it is important to fully evaluate the risk factors for CIN (see Chapter 3). On the other hand, when contrast-enhanced CT is performed in CKD patients with an eGFR < 30 mL/min/1.73 m^2^, it is recommended that the risk of CIN onset be explained and appropriate preventive measures be taken as necessary.

**Level of Evidence: IVa/Grade of Recommendation: B**

**(Minds 2017) Quality of evidence: B/Strength of recommendation: 2**

**Rationale CQ6-1**

Conventionally, the frequency of CIN onset in contrast-enhanced CT has been reported as 6.4% on average (range is 0–25%) [145], but few studies have compared the onset between a contrast media-administered group and a non-administered group. Hence, the effect of contrast media on CIN onset has not been accurately assessed. As background, there were a number of cases that satisfied the diagnostic criteria for CIN due to natural fluctuations of SCr, including patients who did not receive contrast media [145].

After the publication of the CIN guideline 2012, a number of large-scale researches have been published that retrospectively analyzed the risk factors for CIN by applying strict statistical methods and using non-contrast media groups as controls, and they showed that the risk of developing CIN with intravenous administration of contrast media was lower than previously thought [24, 102, 146–158].

It was reported that there was no statistically significant difference in the incidence of AKI between 938 CKD patients receiving contrast-enhanced CT and 1164 CKD patients who did not receive contrast media [146]. Davenport et al. analyzed 8826 patients who were administered contrast media and 8826 who were not with small SCr fluctuations (< 0.3 mg/dL) before CT examination by propensity score matching and showed that contrast media was not a risk factor for CIN in patients with an eGFR ≥ 30 mL/min/1.73 m^2^, but was a risk factor in patients with an eGFR < 30 mL/min/1.73 m^2^ (OR 2.96, 95% CI 1.22–7.17) [147]. In contrast, McDonald et al. analyzed a contrast agent-administered group (12,508 patients) by propensity score matching with a non-administered group and found that contrast media was not a risk factor for CIN in patients with an eGFR < 30 mL/min/1.73 m^2^ (OR 0.97, 95% CI 0.72–1.30) [150]. In both reports, contrast-enhanced CT was not a risk factor for developing CIN in patients with an eGFR ≥ 30 mL/min/1.73 m^2^, though their findings diverged in patients with an eGFR < 30 mL/min/1.73 m^2^. Differences in patient background, the propensity score model, patient selection criteria, etc., have been suggested as causes of this divergence [159, 160]. In response to these criticisms, McDonald et al. re-analyzed 6902 CKD patients using a propensity score model after adopting strict patient selection criteria. As a result, even in patients with an eGFR < 30 mL/min/1.73 m^2^, contrast-enhanced CT was not a risk factor for CIN (OR 1.02, 95% CI 0.63–1.41), dialysis initiation within 30 days (OR 2.33, 95% CI 0.98–3.68), or mortality within 30 days (OR 0.93, 95% CI 0.57–1.29) [10]. Several studies showing that contrast-enhanced CT was not a risk factor for CIN even in patients with an eGFR < 30 mL/min/1.73 m^2^ have been reported [154, 155, 157].

Based on the above findings, in patients with an eGFR ≥ 30 mL/min/1.73 m^2^, it is unlikely that contrast-enhanced CT is a risk factor for CIN, even in patients with an eGFR < 30 mL/min/1.73 m^2^. However, this evidence is not sufficient, since it is a report from a limited facility. Therefore, at present, when conducting contrast-enhanced CT for CKD patients with an eGFR < 30 mL/min/1.73 m^2^, it is necessary to undertake sufficient explanation of CIN and appropriate preventive measures. Even in patients with an eGFR ≥ 30 mL/min/1.73 m^2^, it is still important to fully evaluate the risk factors (see Chapter 3) and take preventive measures as necessary. With regard to the timing of eGFR measurement, the ESUR has recommended measurement within 7 days in patients with acute disease, inpatients, and patients at high risk of CIN and within 3 months in patients with stable renal function [4, 5].

### 6.2 **CQ6-2** Does the risk of developing CIN increase in intensive care patients and severe emergency outpatients after contrast-enhanced CT?

Answer:

In intensive care and severe emergency outpatient patients, there is little evidence that contrast-enhanced CT is a risk factor for developing CIN. However, in these patients, the risk of developing AKI is high irrespective of the administration of contrast media. Therefore, when contrast-enhanced CT is performed, it is recommended to sufficiently explain AKI and CIN and to take appropriate preventive measures.

**Level of Evidence: V/Grade of Recommendation: not applicable**

**(Minds 2017) Quality of evidence: B/Strength of recommendation: 2**

**Rationale CQ6-2**

Contrast-enhanced CT is one of the indispensable examinations in intensive care units and emergency outpatient clinics. Patients in intensive care units or emergency outpatients with acute severe disease are at high risk of developing AKI irrespective of the administration of contrast media; in particular, the frequency of AKI in intensive care patients has been reported to be around 20–50% [161–164]. Therefore, when a patient in an intensive care unit develops AKI after contrast-enhanced CT, it is very difficult to determine whether it has been caused by contrast media or not. In recent years, several reports have been conducted to verify the risk of developing CIN in intensive care and emergency outpatient patients [102, 152, 154, 156, 164–170].

McDonald et al. analyzed 6877 intensive care unit patients using propensity score matching between a contrast agent administration group and a non-administration group and showed no significant difference in the rate of CIN (31% vs. 34%, OR 0.88, 95% CI 0.75–1.05), initiation of dialysis within 7 days (2.0% vs. 1.7%, OR 1.20, 95% CI 0.66–2.17), or death (12% vs. 14%, OR 0.87, 95% CI 0.69–1.10) in patients with an eGFR ≥ 45 mL/min/1.73 m^2^. On the other hand, in patients with an eGFR < 45 mL/min/1.73 m^2^, there were no significant differences in the incidence of CIN (50% vs. 45%, OR 1.21, 95% CI 0.87–1.68) and mortality rate (21% vs. 17%, OR 1.23, 95% CI 0.82–1.83), but the rate of dialysis initiation within 7 days was significantly higher in the contrast agent-administered group (6.7% vs. 2.5%, OR 2.72, 95% CI 1.14–6.46) [154]. In addition, Fukushima et al. retrospectively analyzed 216 CKD patients who received contrast-enhanced CT and reported that cardiac function decline (OR 6.54, 95% CI 1.09–39.3) and entry into the intensive care unit (OR 11.5, 95% CI 2.05–64.1) were significant risk factors for CIN onset [171]. From these reports, compared to general patients, severe disease patients such as those in intensive care units are at higher risk of developing AKI irrespective of the administration of contrast media, especially those patients with an eGFR < 45 mL/min/1.73 m^2^. It is necessary to explain and take countermeasures regarding post-contrast AKI and dialysis initiation in patients in the intensive care unit with an eGFR < 45 mL/min/1.73 m^2^.

In emergency outpatient clinics, patients’ conditions range from mild to severe and many patients entering the intensive care unit are also there. Regarding the CIN risk in contrast-enhanced CT in emergency outpatient clinical practice, Hinson et al. analyzed 16,801 emergency outpatients by propensity score matching among a contrast-enhanced CT group, non-contrast-enhanced CT group, and non-CT-treated group [102]. As a result, it was reported that the frequency of CIN was 6.8% in the contrast-enhanced CT group, 8.9% in the non-contrast-enhanced CT group, and 8.1% in the non-CT-treated group, suggesting that the risk of CIN does not increase with administration of contrast media. Furthermore, even in a sub-analysis in patients with an eGFR < 30 mL/min/1.73 m^2^, an association between contrast media administration and CIN could not be found. Aycock et al. conducted a meta-analysis of research that examined the CIN risk in contrast-enhanced CT with a contrast media non-administered group as the control [156]. The meta-analysis examined 28 articles, including 6 articles on emergency outpatients, and demonstrated that contrast-enhanced CT is not a risk factor for CIN (OR 0.94, 95% CI 0.82–1.07), initiation of dialysis (OR 0.83, 95% CI 0.59–1.16), or death (OR 1.0, 95% CI 0.73–1.36) [156]. From these reports, there is little evidence that contrast-enhanced CT is a risk factor for the development of CIN in emergency outpatients. However, since renal function and risk factors in emergency outpatients are often unknown and their disease severities also vary, it is important to take appropriate preventive measures, especially in seriously ill patients.

### 6.3 **CQ6-3** Does reduction of the contrast media volume in contrast-enhanced CT decrease the risk of developing CIN?

Answer:

There is a possibility that a reduction in the contrast media volume in contrast-enhanced CT may reduce the risk of developing CIN. For patients with a high CIN risk (CQ 6-1, 2) in particular, it is recommended to utilize the minimum amount of contrast media necessary for diagnostic efficacy.

**Level of Evidence: IVa/Grade of Recommendation: C1**

**(Minds 2017) Quality of evidence: C/Strength of recommendation: 2**

**Rationale CQ6-3**

It is strongly recommended to minimize the amount of the contrast media used in CAG, in which the contrast media is administered intra-arterially, as it reduces the risk of developing CIN (CQ 5-2). Meanwhile, although there are few reports on the relationship between contrast media volume and CIN in contrast-enhanced CT via intravenous administration, Weisbord et al. analyzed 421 CKD patients with an eGFR 30–59 mL/min/1.73 m^2^ who received contrast-enhanced CT and showed that CIN risk rises when the contrast media usage exceeds 100 mL (contrast agent concentration unknown) (OR 3.3, 95% CI 1.0–11.5) [54]. Recently, Jochheim et al. examined 361 patients with advanced aortic valve stenosis who received contrast-enhanced CT before TAVR and demonstrated that CIN was found in 10.5% of patients, the prevalence of CKD (eGFR < 60 mL/min/1.73 m^2^) was higher in the CIN group than in the non-CIN group (81.6% vs. 64.4%), and the amount of contrast media used tended to be large [172]. Multivariate analysis showed that neither eGFR nor contrast agent usage alone was a risk factor for CIN but that “eGFR x contrast media usage” was a significant risk factor (OR 2.70, 95% CI 1.33–5.49). In addition, sub-analysis showed that in patients with an eGFR < 60 mL/min/1.73 m^2^, the incidence of CIN was significantly higher in patients in whom > 90 mL of contrast media was used than in patients who received < 90 mL. On the other hand, in patients with an eGFR ≥ 60 mL/min/1.73 m^2^, there was no association between contrast medium usage and the onset of CIN. Since this was a study on pre-TAVR patients with a high CIN risk, older age (mean age, 81 years), and complications from comorbidities (such as coronary artery disease, diabetes, hypertension, anemia, etc.), care must be taken in interpreting the results. Based on the above reports, there is a possibility that the risk of developing CIN may be lowered by decreasing the contrast media volume in contrast-enhanced CT, but it is difficult to say whether the evidence level is sufficient. Although safe contrast media usage is not uniformly defined, in view of the above reports, in patients with a high CIN risk (CQ 6-1, 2), usage exceeding 90–100 mL should be avoided. On the other hand, excessive reduction of contrast media may lower the diagnostic accuracy; therefore, the amount of contrast media to be used should be determined in consideration of the inherent risks and specific benefit of the examination for each patient. Moreover, it is obvious that sufficient explanation and countermeasures should be undertaken to prevent CIN and that after examination, the patient’s renal function and condition should be carefully evaluated.

The American College of Radiology, a major international academic opinion on the minimization of contrast media in patients with a high CIN risk, states that “there is little evidence that reduction of contrast media reduces the risk of CIN in intravenous administration”, and they do not recommend that the volume of contrast media be reduced [142]. The ESUR recommends to “use the lowest dose of contrast media necessary for diagnosis in patients with CIN risk” [157, 158]. The Society of Cardiovascular Computed Tomography also recommends that the amount of contrast agent be reduced in patients with a CIN risk [173].

**Fig. 3:**
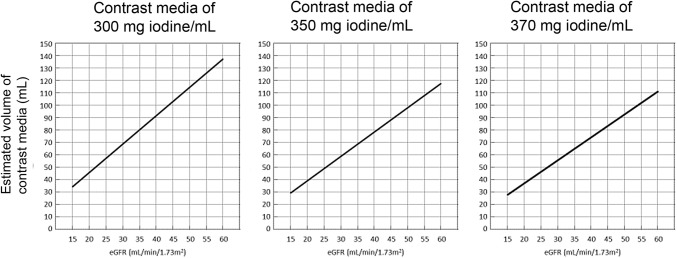
Volumes of contrast media associated with a 5% incidence of CIN. CIN was defined as an increase in SCr level by 44.2 mmol/L (0.5 mg/dL) or ≥ 20 to 25% within 48–72 h after contrast exposure. The formula used to calculate the volume of contrast media associated with CIN has been validated in only 1 study by Nyman et al. [108], and there is insufficient evidence supporting the formula. Readers should be aware of this and should use these data for reference only. The formula was developed on the basis of data of patients undergoing cardiac catheterization rather than CT. *CIN* contrast-induced nephropathy, *CT* computed tomography, *eGFR* estimated glomerular filtration rate

When contrast-enhanced CT is performed with a reduced volume of contrast media, it is recommended to use low tube voltage imaging and iterative reconstruction when possible to increase contrast enhancement and prevent image quality deterioration (CQ 6-4). Using a formula described by Nyman et al. [174], the volumes of contrast media that are associated with the 5% incidence rate of CIN in patients with various eGFRs can be calculated (Fig. 3). This formula has been validated in only a few studies [108], and sufficient evidence in support of the formula is not available. Readers should be aware of this and should only use these data as a reference.

### 6.4 **CQ6-4** Is there a recommended scan method for decreasing contrast media usage in contrast-enhanced CT?

Answer:

When decreasing the contrast media usage, it is recommended to combine low tube voltage imaging and iterative reconstruction in facilities where it is possible.

**(Minds 2017) Quality of evidence: A/Strength of recommendation: 1**

**Rationale CQ6-4**

Often, CT examination is conducted at a tube voltage of 120 kV. Low tube voltage scanning is conducted using tube voltages that are lower than the standard 120 kV (e.g., 80 kV, 100 kV, etc.) [175]. The use of low tube voltage increases the effect of enhancement conferred by the iodinated contrast media owing to the contribution of the increased photoelectric effect to X-ray attenuation and the increased CT value of iodine (atomic number 53). Although there is some variation depending on the CT scanners, the contrast of the iodinated contrast agent increases by about 25% at 100 kV and 70% at 80 kV compared to the normal 120 kV. That is, even if the contrast media is reduced by about 20% at 100 kV and 40% at 80 kV, the same contrast effect as at the standard 120 kV can be obtained. However, scanning with a low tube voltage may result in the deterioration of image quality (increase in image noise and artifacts) due to an insufficient dose of x-ray photons for generating an image. Therefore, when performing low tube voltage imaging, it is necessary to set the tube current (mA) higher to compensate for the shortage of X-ray photons [176]. However, as there is a limit to the tube current output of the CT systems and the X-ray tube load is large at the high current setting, it is generally difficult to use.

Recently, a new CT image reconstruction algorithm (iterative reconstruction: IR) has become widespread, replacing the conventional filtered back projection method (FBP). The IR method is characterized by reducing image noise and artifacts and improving image quality compared with the FBP method [177]. Combined use of IR and low tube voltage imaging can improve image quality degradation due to X-ray photon deficiency [175, 178–187].

When switching to low tube voltage imaging to reduce the contrast agent volume, it is common to adjust the tube current so that the volume CT dose index will be at the same level as the standard voltage scan (120-kV scan) in order to ensure image quality. There are many types of IR with various manufacturers and generations and differing characteristics [188]. In addition, adjustment of the reconstruction parameters is required according to the contrast of the object to be examined (e.g., high-contrast objects like CT angiography or low-contrast objects like a liver metastasis survey). Therefore, in order to use the combination of low tube voltage scan and IR effectively, the scanning parameters must be verified and optimized beforehand. Even when IR is used, image quality degradation may occur in an 80-kV scan in patients with a large body size; therefore, in general, 80-kV imaging should only be applied to non-obese patients.

In recent years, a contrast media reduction method using virtual monochromatic X-ray imaging by dual energy CT has been reported [189–194] in which virtual monochromatic X-ray images are generated based on dual energy data and it is possible to virtually represent a CT image of an arbitrary single energy level [effective energy (keV)]. The lower the single energy, the higher the CT value of the iodine contrast media. Nagayama et al. reported that the contrast media volume could be reduced by 50% without deteriorating the image quality by using a virtual monochromatic X-ray CT (40–55 keV) [194]. At present, the dissemination of dual energy CT is limited and the technique is still being developed, and an accumulation of further evidence accumulation is necessary to validate its use.

### 6.5 **CQ6-5** Does repeated contrast-enhanced CT at short intervals increase the risk of developing CIN?

Answer:

Since repeated contrast-enhanced CT at short intervals may increase the risk of developing CIN, we do not recommend performing a repeat contrast-enhanced CT within 24–48 h of the first.

**Level of Evidence: IVa/Grade of Recommendation: C2**

**Rationale CQ6-5**

Abujudeh et al. reported that CIN occurred in 21 (12.8%) of 164 patients who underwent contrast-enhanced CT twice within 24 h [195]. Since this incidence is higher than that of general CIN, it is suggested that the onset of CIN may increase in the context of repeated contrast-enhanced CT. Moreover, Trivedi et al. examined 28 patients who received contrast media twice and found that SCr statistically significantly increased after the second injection of contrast media, and 4 out of 28 patients (14.3%) developed CIN more frequently than after first injection [196]. Hong et al. demonstrated that 66 patients (8.0%) developed CIN among 820 cancer-bearing patients who received contrast-enhanced CT and that repeated contrast-enhanced CT within 72 h was a risk factor for CIN (OR 4.1, 95% CI 1.3–12.6) [197].

Conversely, in a study of stroke patients who underwent contrast-enhanced CT, Hopyan et al. reported that no one developed CIN among 55 patients who underwent a second imaging examination within 24 h [198]. Furthermore, in a study on acute intracranial hemorrhage patients, it was reported that there was no association between the number of contrast-enhanced CTs and CIN onset [199]. In addition, a report showed that there was no difference in the frequency of AKI development between the group to whom contrast media were administered twice within 32 h and the control groups who did not receive contrast media [200], while yet another reported that the frequency of CIN did not increase even when angiography was performed after contrast-enhanced CT [201]. The scientific basis of the concept that the risk of CIN increases with a repeated contrast-enhanced CT in the short-term remains insufficient. However, short-term repetitive examinations of contrast-enhanced CT should be avoided in principle, as there is the possibility of an increased CIN risk. Patients who inevitably must undergo multiple contrast examinations in a short period of time should receive careful explanation of CIN and appropriate preventive measures, and strict observation of the changes of renal function and general condition over time after the examination is critical. In terms of the perspective of a major academy concerning the risks conferred by the short-term repetition of contrast-enhanced CT, the American College of Radiology claims “There is not enough evidence as a basis for avoiding short-term repetitive contrast CT” [179], while the ESUR states “It poses a risk for CIN to repeat contrast agent administration within 48–72 h” [4, 5].

## 7. Prevention of CIN; hydration therapy

### 7.1 **CQ7-1** Does physiological saline hydration decrease the risk of developing CIN?

Answer:

1. We recommend using physiological saline intravenously before and after contrast-enhanced examination in CKD patients, as they are at high risk of developing CIN.

**Level of Evidence: II/Grade of Recommendation: A**

2. We recommend using isotonic solutions to prevent CIN because isotonic 0.9% sodium chloride (physiological saline) is superior to hypotonic 0.45% sodium chloride in preventing CIN.

**Level of Evidence: II/Grade of Recommendation: A**

**Rationale CQ7-1**

In the 1980s, Eisenberg et al. [202, 203] demonstrated that the development of CIN in patients with CKD undergoing contrast-enhanced examination cold be prevented by intravenous administration of physiological saline during the examination. Trivedi et al. [204] conducted an RCT to assess the role of saline hydration in the development of CIN. A total of 53 patients with normal kidney function who were set to undergo non-emergency cardiac catheterization were randomized to a group of patients receiving normal saline intravenously or a group of patients who were allowed unrestricted oral fluids. CIN (defined as an increase in SCr levels of ≥ 0.5 mg/dL within 48 h of contrast exposure) developed in 1 of the 27 patients (3.7%) receiving physiological saline infusion and 9 of the 26 patients (34.6%) with unrestricted oral fluids (p = 0.005), indicating that physiological saline hydration significantly decreases the incidence of CIN. In the RENO Study, 111 patients with ACS undergoing emergency PCI were randomly assigned to receive an initial intravenous bolus of 5 mL/kg/h of alkaline saline solution with 154 mEq/L of sodium bicarbonate over 1 h before PCI (group A) or to receive physiological saline hydration after PCI (group B) [205]. The incidence of CIN was 1.8% in group A and 21.8% in group B (p = 0.032). In emergency PCI cases targeting ST elevation myocardial infarction patients, it was reported that administration of physiological saline from the beginning of PCI to 24 h after suppresses the onset of CIN [206], suggesting that administration of physiological saline alone after using contrast media may facilitate the prevention of CIN. According to these findings, it is recommended that patients receive intravenous physiological saline before and after contrast media exposure to prevent CIN.

In an RCT comparing the effects of isotonic and hypotonic fluids on the incidence of CIN, the isotonic solution (0.9% physiological saline) was superior to the hypotonic solution (0.45% sodium chloride) [207]. In this study, 1620 patients scheduled for selective or emergency coronary angioplasty were randomly assigned to receive isotonic (n = 809) or hypotonic (n = 811) hydration prior to intervention. The incidence of CIN (defined as an increase in SCr levels of ≥ 0.5 mg/dL within 48 h) was significantly reduced with isotonic (0.7%, 95% CI 0.1–1.4%) vs. hypotonic (2.0%, 95% CI 1.0–3.1%) hydration (p = 0.04). Many patients had normal kidney function at baseline, and non-ionic low-osmolar contrast media were used.

Since these findings support the efficacy of isotonic fluids, such as physiological saline, in the prevention of CIN, we recommend their use as a preventive measure for CIN. The volume of isotonic fluids infused should be adjusted according to the cardiac function and general condition of the patient. The use of isotonic fluids to prevent CIN should be considered for patients with an eGFR of < 30 mL/min/1.73 m [2] who are undergoing intravenous administration of contrast media such as contrast-enhanced CT, for intensive care patients and severe emergency outpatients with an eGFR of < 45 mL/min/1.73 m^2^, and for patients with an eGFR of < 60 mL/min/1.73 m^2^ who are undergoing intra-arterial administration of contrast media such as CAG. However, in one randomized controlled study [208] of patients with an eGFR of 30-59 mL/min/1.73 m^2^ who received contrast media, there was no difference in incidence of CIN between the patients who received physiological saline infusion and those who did not. Further study of renal function as an indication for infusion is warranted.

### 7.2 **CQ7-2** Does oral water intake decrease the risk of developing CIN?

Answer:

There is no sufficient evidence that oral water intake is as effective as intravenous hydration therapy in preventing the development of CIN. We recommend that patients receive hydration therapy or other established preventive measures rather than relying on oral water intake to prevent CIN.

**Level of Evidence: II/Grade of Recommendation: C1**

**Rationale CQ7-2**

It is difficult to conduct intravenous hydration as a measure to prevent CIN in outpatients or patients undergoing emergency imaging. In such patients, oral fluid loading to prevent dehydration and promote diuresis has been attempted. Trivedi et al. [204] evaluated the effects of unrestricted oral fluids and intravenous saline hydration on the incidence of CIN in patients undergoing non-emergency cardiac catheterization and reported that saline hydration was superior to oral fluids in terms of the prevention of CIN and severity of kidney dysfunction. Conversely, in a study targeting patients with relatively normal renal function, it was reported that oral water intake was not inferior to infusion in preventing CIN. In a randomized study of the effects of oral hydration with mineral water versus intravenous hydration with isotonic solution on kidney function in patients with diabetes undergoing elective CAG and PCI [209], 52 patients (group 1; mean CCr: 70.3 mL/min) were hydrated intravenously (1 mL/kg/h) with isotonic solution (0.9% NaCl) during the 6 h before and the 12 h after CABG or PCI, and 50 patients (group 2; mean CCr 79 mL/min) received oral water intake (1 mL/kg/h) during the 6–12 h before and the 12 h after CAG or PCI. At 72 h after the procedure, the mean CCr was 65.3 mL/min in group 1 and 73.5 mL/min in group 2 [not significant (NS)]. The incidence of CIN was 5.77% in group 1 and 4.00% in group 2 (NS). In a different study, patients with CKD stage G1-2 undergoing CAG or PCI were divided into one group who drank tap water as much as possible 12 h before and after and another group who received physiological saline (1 mL/kg/h) 12 h before and after. The incidence of CIN was 6.9% in the drinking water group and 7.3% in the physiological saline group, and no significant difference was found between the two groups [210]. In a study on patients with normal renal function (SCr < 110 μmol/L ≈ 1.24 mg/dL) undergoing scheduled CAG or PCI, the preventive effect of the administration of 1 mL/kg/h of physiological saline solution 12 h before and 24 h after the procedure on CIN onset was compared to two drinking load groups (one group who received 500 mL of tap water for 2 h before and 2000 mL for 24 h after the procedure and one group who received 2000 mL of tap water only for 24 h after the procedure). The CIN incidence rates were 5.0, 7.5, and 5.0%, respectively, and no significant difference was found among the 3 groups [211]. In the PREPARED study, 36 patients with CKD (SCr ≥ 1.4 mg/dL) undergoing elective cardiac catheterization were randomized to receive either an outpatient hydration protocol including pre-catheterization oral hydration (1000 mL oral water intake over 10 h) followed by 6 h of intravenous hydration (0.45% sodium chloride at 300 mL/h; n = 18) beginning just before contrast exposure or overnight intravenous hydration (0.45% sodium chloride at 75 mL/h for 12 h before and after the catheterization procedures; n = 18) [212]. The maximal changes in SCr levels in the inpatient (0.21 ± 0.38 mg/dL) and outpatient (0.12 ± 0.23 mg/dL) groups were similar (NS). They concluded that an oral hydration strategy prior to PCI/CAG was similar to intravenous hydration in preventing contrast-associated changes in SCr levels. The results of the earlier-described RCT suggest that oral hydration prior to PCI/CAG may be effective in the prevention of CIN. One RCT investigated whether oral intake of sodium chloride and water exerts effects similar to that of intravenous saline hydration [213]. In that study of the use of saline hydration to prevent CIN in 312 patients with CKD (mean CCr: 37 mL/min/1.73 m^2^), patients were randomly assigned. In the oral group, 76 patients received 1 g/10 kg of body weight of sodium chloride orally for 2 days before the procedure. In the intravenous group, 77 patients received 0.9% saline intravenously at a rate of 15 mL/kg for 6 h before the procedure. The incidence of CIN was 6.6% in the first group and 5.2% in the second group (NS). The authors concluded that oral sodium chloride intake was as effective as intravenous saline hydration for the prevention of CIN. Despite these reports indicating that oral hydration and intravenous saline infusion are similar in terms of the prevention of CIN, there is no conclusive evidence supporting the efficacy of oral hydration at this time. Oral hydration with water cannot be recommended as an alternative to intravenous infusion of physiological saline. Further studies are needed to confirm whether CIN can be prevented by oral water intake prior to the procedure and intravenous hydration after the procedure in patients in whom preprocedural intravenous hydration is not feasible. There is no conclusive evidence regarding the equivalence of oral sodium chloride intake and intravenous saline hydration in the prevention of CIN. However, oral hydration prior to contrast exposure is recommended as a measure to treat dehydration and prevent discomfort caused by contrast media.

### 7.3 **CQ7-3** Does sodium bicarbonate-based hydration decrease the risk of developing CIN?

Answer:

Sodium bicarbonate-based hydration may decrease the risk of developing CIN. When infusion time is limited, administration of sodium bicarbonate-based hydration is recommended.

**Level of Evidence: I/Grade of Recommendation: B**

**Rationale CQ7-3**

The efficacy of sodium bicarbonate-based hydration in the prevention of CIN has been evaluated. Seven meta-analyses have been published on the comparison of sodium bicarbonate-based hydration with physiological saline hydration in the prevention of CIN, and all but 1 analysis concluded that sodium bicarbonate-based hydration was superior to saline hydration in reducing the risk of CIN [214–221]. In 2009, Zoungas et al. [214] searched data published from 1950 to 2008 and reviewed 23 published and unpublished RCTs of intravenous sodium bicarbonate (9 peer-reviewed studies and 14 abstracts) with data on 3563 patients. They reported that the pooled relative risk of CIN in patients receiving sodium bicarbonate-based hydration was 0.62 (95% CI 0.45–0.86). Other meta-analyses yielded similar results in terms of the prevention of CIN using sodium bicarbonate-based hydration. However, no significant differences between sodium bicarbonate-based hydration and saline hydration were observed in terms of the incidence of hemodialysis, incidence of heart failure, or mortality. They concluded that sodium bicarbonate-based hydration may decrease the incidence of CIN but does not differ from saline hydration in terms of kidney function and hard endpoints. Researchers have pointed out that the studies included in these meta-analyses differ substantially in design and that sodium bicarbonate-based hydration was reported to be effective in many published articles and ineffective in other studies published as abstracts only.

In a meta-analysis of 14 studies (3 large and 11 small studies) of 2290 patients, there was no evidence of a benefit for hydration with sodium bicarbonate compared to sodium chloride for the prevention of CIN in the large trials [221]. The report pointed out that including studies of lower methodological quality in the analysis may have led to a biased conclusion. Therefore, the researchers performed a subsequent analysis limited to 8 studies that met the quality criteria, including over 100 patients, and a similar dose and strategy between treatment groups if *N*-acetylcysteine (NAC) use was permitted. The relative risk for sodium bicarbonate (n = 945) compared with that for sodium chloride (n = 945) was 0.71 (95% CI 0.41–1.03), which was not statistically significant but was suggestive of a superior efficacy of the sodium bicarbonate-based hydration.

Readers of these meta-analyses should be aware that a typical protocol of sodium bicarbonate-based hydration consists of an infusion of about 150 mEq/L solution at 3 mL/kg/h for 1 h before and an infusion at 1 mL/kg/h for 6 h after contrast exposure and varies in duration from a typical protocol of saline hydration with a 6 to 12-h infusion at 1 mL/kg/h before and after contrast exposure. In these meta-analyses, data were not adjusted for the difference in the duration of infusion. In addition, preprocedural hemofiltration, which is one way to achieve alkalization, has been reported to be effective for preventing CIN, and alkalization is also considered effective in the prevention of CIN. Indeed, Tamai et al. compared 2 different concentrations of bicarbonate (833 mEq/L vs. 160 mEq/L) with the same protocol (3 mL/kg/h for 1 h before and 1 mL/kg/h for 7 h after imaging) and reported that the incidence of CIN was lower when a high-concentration sodium bicarbonate infusion was used [222]. However, in a study of patients randomized to receive either sodium chloride or sodium bicarbonate administered at the same rate (3 mL/kg for 1 h before CAG, decreased to 1.5 mL/kg/h during the procedure and for 4 h after the completion of the procedure), the incidence of CIN did not differ between the 2 groups [223]. Since 2009, 7 reports with differing designs have been published on the use of sodium bicarbonate-based hydration. Sodium bicarbonate-based hydration was concluded to be effective in 3 studies [224–226] and ineffective in 4 studies [227–230]. There have been 3 reports on sodium bicarbonate-based hydration in Japan. Ueda et al. [224] compared bolus saline infusion with bolus sodium bicarbonate infusion immediately before emergency PCI and reported that sodium bicarbonate infusion significantly decreased the incidence of CIN by 88% (RR: 0.128, 95% CI 0.016–0.91, p = 0.01). In an RCT of 144 patients with mild CKD undergoing elective CAG, Tamura et al. [225] reported that the incidence of CIN was lower in patients who received standard saline hydration (12 h before contrast exposure) plus a single-bolus intravenous administration of 20 mEq/L sodium bicarbonate (MEYLON 20 mL) immediately before contrast exposure than in patients who received standard saline hydration alone (p = 0.017). Motohiro et al. [226] conducted an RCT in 155 patients and reported that the incidence of CIN in patients undergoing CAG was significantly lower in 78 patients who received 3 h of saline hydration followed by 3 h of sodium bicarbonate-based hydration at 1 mL/kg/h prior to CAG and 6 h of sodium bicarbonate-based hydration after CAG than in 77 patients who received saline hydration alone (p = 0.012). In the PREVENT study conducted in Korea, 382 patients with diabetes and CKD were randomly assigned to receive saline hydration at 1 mL/kg/h for 12 h before and after CAG or PCI (saline group, n = 189) or sodium bicarbonate at 3 mL/kg/h for 1 h before contrast exposure and at 1 mL/kg/h from the initiation of the procedure to 6 h after the procedure (bicarbonate group, n = 193) [227]. All patients received oral NAC 1200 mg twice daily for 2 days. The incidence of CIN was 5.3% in the saline group and 9.0% in the bicarbonate group, but the difference was not significant (p = 0.17).

Even in reports published after 2011, the effectiveness of sodium bicarbonate on CIN prevention is limited compared to that of physiological saline solution. We have separately compiled the studies involving emergency cases with limited infusion time and studies on scheduled examinations where ample infusion time can be secured. Among the emergency case reports, Maioli et al. compared a group treated with physiological saline (1 mL/kg/h) for 12 h only after using contrast media and a group treated with 154 mEq/L bicarbonate infusion for 1 h (3 mL/kg/h) before and 12 h (1 mL/k/h) after using a contrast medium [231] and reported that CIN onset was significantly lower in the bicarbonate infusion group (12%) than in the physiological saline infusion group (22.7%). However, in that study, the physiological saline infusion was not administered before the use of contrast media and it is insufficient as evidence that the sodium bicarbonate is more effective than the physiological saline. Manari et al. [232] compared 4 groups in terms of CIN onset: physiological saline infusion for 1 h (1 mL/kg/h and 3 mL/kg/h) before using contrast media, for 11 h (1 mL/kg/h) after, 154 mEq/L bicarbonate infusion for 1 h before using contrast media (1 mL/kg/h and 3 mL/kg/h), and for 11 h (1 mL/kg/h) after. In addition, Gomes et al. [233] also compared physiological saline infusion and 154 mEq/L bicarbonate at 3 mL/kg/h for 1 h before and 1 mL/kg/h for 6 h after the use of the contrast media. In these studies, CIN onset between the physiological saline and sodium bicarbonate groups was not different. Therefore, whether sodium bicarbonate can suppress CIN onset comparably to physiological saline infusion when used for a limited time in emergency cases remains inconclusive.

The results are also similar in scheduled examinations where contrast media were used. Boucek et al. [234] used exactly the same protocol as Gomes et al. [233] and showed that rate of CIN onset between physiological saline and 154 mEq/L bicarbonate was not different. In addition, Solomon et al. [235] compared physiological saline with 154 mEq/L bicarbonate at 5 mL/kg/h for 1 h before and at 1.5 mL/kg/h for 4 h after the use of contrast media and demonstrated no significant difference in the onset of CIN between the 2 groups. Klima et al. [236] suggested that physiological saline is superior for the prevention of CIN onset in scheduled examinations. Physiological saline infusion at 1 mL/kg/h for 12 h before and after the use of contrast media was compared with 166 mEq/L of bicarbonate infusion at 3 mL/kg/h for 1 h before and at 1 mL/kg/h for 6 h after the contrast media. The CIN incidence was significantly higher in the bicarbonate group (9%) than in the physiological saline group (1%). In addition, 5177 patients with an eGFR 15–59.9 mL/min/1.73 m^2^ who were scheduled to undergo angiography were divided into 4 groups (2 × 2; physiological vs. sodium bicarbonate and NAC vs. placebo) [237], and no significant difference between physiological saline and sodium bicarbonate was observed in terms of CIN onset.

These findings suggest that sodium bicarbonate may be superior to saline in the prevention of CIN in patients who have only a limited time to receive intravenous infusion (e.g., patients requiring emergency care). However, sodium bicarbonate-based hydration does not significantly decrease the risks of hemodialysis and death. Therefore, we cannot conclude that bicarbonate-based hydration is definitively more effective than physiological saline.

### 7.4 **CQ7-4** Is short-term intravenous sodium bicarbonate hydration as effective as standard intravenous hydration in preventing CIN?

Answer:

There is no conclusive evidence that short-term intravenous sodium bicarbonate hydration is as effective as standard intravenous hydration for preventing CIN. Excluding emergency cases with limited infusion time, it is recommended to administer infusion for an extended period of time.

**Level of Evidence: II/Grade of Recommendation: C2**

**Rationale CQ7-4**

It is difficult to conduct RCTs comparing short-term intravenous hydration (e.g., 1-h intravenous hydration before contrast exposure) with standard intravenous hydration because short-term intravenous hydration is required only for patients undergoing emergency PCI. In an RCT of 63 patients with CKD who received either 12-h intravenous hydration at 1 mL/kg/h or bolus hydration at a volume of 250 mL over 1 h immediately before the angiography, the incidence of CIN was 0% in patients receiving overnight hydration and 10.8% in patients receiving bolus hydration [238]. Meanwhile, in a study comparing intravenous hydration of 2000 mL/day within 12 h before and after contrast exposure and volume expansion with 300 mL saline immediately before the administration of contrast media, the incidence of CIN did not differ between the groups [239]. Among 4 RCTs comparing 1-h sodium bicarbonate hydration at 3 mL/kg/h with 612-h saline hydration at 1 mL/kg/h, 3 RCTs did not show a difference in the incidence of CIN between the groups [227, 230, 240]. These findings suggest that short-term sodium bicarbonate-based hydration is as effective as standard saline hydration in preventing CIN. The RENO study [205] and the REMEDIAL study [241] showed that administration of sodium bicarbonate for 1 h before using contrast media can suppress the onset of CIN.

The benefit of short-term sodium bicarbonate infusion in comparison with physiological saline has not been confirmed. Clinical trials comparing the effectiveness of sodium bicarbonate infusion for 1 h before using contrast media with physiological saline for 12 h are limited and the results are insufficient as evidence. Future investigations are warranted.

## 8. Prevention of CIN: pharmacologic therapy

Proposed mechanisms of CIN include a decrease in renal blood flow, hypoxia of the renal medulla due to vascular constriction, and kidney injury due to reactive oxygen species (ROS). Thus, it has been expected that drugs exerting antioxidant effects as well as drugs that dilate blood vessels may prevent or mitigate CIN, and many clinical studies of these drugs have been conducted. Most studies have targeted patients undergoing CAG, and studies targeting patients who received intravenous contrast media, such as enhanced CT, are scarce. Moreover, there are no established pharmacologic preventive strategies. The ESUR guideline on contrast media, which was published after the last meeting of this committee, stated that no pharmacological prophylaxis has yet been shown to offer consistent protection against CIN.

### 8.1 **CQ8-1** Does the administration of NAC prevent CIN onset?

Answer:

We do not recommend the use of NAC for the prevention of CIN onset.

**Level of Evidence: I/Grade of Recommendation: C2**

**Rationale CQ 8-1**

Proposed mechanisms of CIN include a decrease in renal blood flow, hypoxia of the renal medulla due to vascular constriction, and kidney injury due to ROS. Accordingly, it has been expected that CIN may be prevented by drugs exerting antioxidant effects such as NAC, ascorbic acid, sodium bicarbonate, and statins, as well as drugs that dilate blood vessels and increase renal blood flow such as human atrial natriuretic peptide (hANP), dopamine, fenoldopam, prostaglandin, and theophylline, and many clinical studies have been conducted to examine the effect of these drugs on the prevention of CIN. However, no conclusive evidence has been obtained for any of them.

NAC, an antioxidant with vasodilative properties has been proven effective in the treatment of hepatic injury due to acetaminophen and is indicated for the treatment of this condition in Japan and the United States. Animal studies have revealed its protective effect on the myocardium and renal function, and it was expected that NAC could prevent CIN in humans as well. Since the report by Tapel et al. on the effect of NAC (600 mg twice daily, orally) in preventing CIN, many RCTs and meta-analyses have been conducted.

Twenty-one RCTs were conducted between 2011 and 2017 [242–262]. The Agency for Healthcare Research and Quality (AHRQ) conducted a meta-analysis on the preventive strategies for CIN that included 54 RCTs investigating the use of NAC with intravenous saline versus intravenous saline with or without a placebo [263]. In the comparison between NAC plus intravenous saline versus intravenous saline alone, NAC reduced the incidence of CIN with a borderline clinical significance. They concluded that the strength of evidence was low, and most of the studies had at least one important study limitation. Similarly, Subramaniam et al. conducted systematic reviews of 54 RCTs on NAC plus intravenous saline versus intravenous saline alone and concluded that the evidence was not strong enough to support routine use [264]. In Japan, the indication of NAC is acetaminophen-induced hepatic injury, and the use of NAC for the prevention of CIN is off-label. At present, the strength of the evidence concerning the preventive effect of NAC on CIN is low, and we do not recommend routine use of NAC as a preventive strategy.

Future research is needed to clarify the effect of NAC on CIN prevention in high-risk populations.

### 8.2 **CQ 8-2** Does administration of hANP prevent CIN onset?

Answer:

We do not recommend the use of hANP for prevention for CIN onset.

**Level of Evidence: I/Grade of Recommendation: C2**

**Rationale CQ 8-2**

An intrinsic peptide, hANP exerts natriuretic, afferent arteriole dilatation, anti-renin, and anti-aldosterone effects and has been reported to be beneficial in the treatment of AKI after cardiac surgery. Studies examining the effect of hANP on the prevention for CIN have failed. Two additional studies on hANP have been reported from Japan; however, the dose varied among the studies and the results were inconsistent [265, 266]. Three RCTs on brain natriuretic peptide (BNP) for cardiovascular interventions have been published, in which BNP was found to reduce the risk of CIN [267–269]; however, a BNP product is not commercially available currently in Japan. Therefore, we do not recommend the use of hANP for CIN prevention.

### 8.3 **CQ 8-3** Does administration of ascorbic acid prevent CIN onset?

Answer:

We do not recommend the use of ascorbic acid for the prevention of CIN.

**Level of Evidence: I/Grade of Recommendation: C2**

**Rationale CQ 8-3**

Ascorbic acid exerts an antioxidant action against ROS and potentiates the effects of other antioxidants. Ten RCTs on ascorbic acid for CIN prevention have been conducted, among which 6 RCTs were published after 2011 [241, 252, 257, 270–276]. All of the studies targeted patients undergoing cardiovascular interventions, and no study targeted patients who received intravenous contrast media. In addition, the AHRQ conducted a meta-analysis of 8 RCTs and Subramaniam et al. conducted meta-analysis including 6 RCTs [263, 264]. They reported a statistically insignificant reduced risk of CIN, and the strength of evidence was low. Thus, we do not recommend the use of ascorbic acid for CIN prevention.

### 8.4 **CQ 8-4** Does administration of statins prevent CIN onset?

Answer:

We do not recommend the use of statins for the prevention of CIN onset.

**Level of Evidence: I/Grade of Recommendation: C2**

**Rationale CQ 8-4**

Statins exerts pluripotential actions, including antioxidant and anti-inflammatory actions. Eight studies have compared statins with placebo, among which 7 were published after 2011 [277–284]. The target population was patients undergoing cardiovascular interventions, and many studies included patients with normal renal function (eGFR > 60 mL/min/1.73 m^2^). Only 2 studies compared statins with placebo in patients with impaired renal function. Meta-analyses on the effectiveness of statins for patients undergoing cardiovascular interventions showed a risk of bias and need for future studies [263, 264].

At present, as the evidence is insufficiently strong to recommend the routine use of statins for CIN prevention and its use as such is off-label, we do not recommend the use of statins for CIN prevention. However, we do not oppose the use of statins for cardiovascular protection purposes in patients with cardiovascular disease who receive iodinated contrast media.

## 9 Prevention of CIN; blood purification therapy

### 9.1 **CQ9-1** Does blood purification therapy conducted after contrast exposure as a measure to prevent CIN decrease the risk of developing CIN?

Answer:

Blood purification therapy after administering contrast media does not decrease the risk of developing CIN and is not recommended. In particular, hemodialysis therapy is not recommended.

**Level of Evidence: I/Grade of Recommendation: D**

**Rationale CQ9-1**

Cruz et al. reported a meta-analysis [285] analyzing 9 RCTs and 2 non-RCTs on the effect of contrast media removal by blood purification therapy after contrast media administration in preventing the onset of CIN, and they concluded that blood purification therapy did not reduce the risk of CIN onset as compared to the conventional therapy (RR 1.02, 95% CI 0.54–1.93) and that hemodialysis therapy actually increased the risk (RR 1.61, 95% CI 1.13–2.28). The findings of this report validate the statement in the CIN guideline 2012.

Contrast media can be administered in patients receiving maintenance dialysis therapy whose renal function is disrupted if there is no volume overload, including any circulating plasma volume increase due to high osmotic pressure. It is not necessary to commence dialysis after the administration of contrast media [286]. On the other hand, in cases of AKI, contrast media can be carefully used provided there is a possibility of renal function recovery.

## 10 Treatment of CIN

### 10.1 CQ10-1 Dose loop diuretics therapy improve renal recovery after CIN onset ?

Answer:

We recommend against the use of loop diuretics for the treatment of CIN because the evidence that loop diuretic administration after CIN onset suppresses the progression of renal dysfunction is poor, and their use may actually cause harm.

**Level of Evidence: VI/Grade of Recommendation: C2**

**Rationale CQ10-1**

Studies examining the effect of loop diuretics on the treatment of CIN were not found. The CIN guideline 2012 did not recommend the administration of loop diuretics, since no study had specified CIN patients as subjects and no significant effect of loop diuretics was observed even in RCTs that targeted AKI patients. Furthermore, the AKI guideline 2016 [3] stated that loop diuretics should not be administered as a treatment for AKI except for the purpose of correcting fluid overload. After the onset of AKI, renal conditions are no longer suitable for loop diuretics, and their use may increase the risk of AKI progression by decreasing the effective circulating plasma volume. It is important to maintain appropriate body fluid volume and blood pressure, maintain adequate circulation volume in the kidneys and avoid exposure to nephrotoxic substances.

### 10.2 **CQ10-2** Dose hydration therapy improve renal recovery after CIN onset ?

Answer:

Hydration therapy for the treatment of kidney dysfunction after the onset of CIN is not recommended except in patients with intravascular volume depletion.

**Level of Evidence: VI/Grade of Recommendation: C2**

**Rationale CQ10-2**

Clinical trials examining the effect of hydration therapy on patients after the onset of CIN were not found. The AKI guideline 2016 [3] recommends considering the possibility of renal AKI that is resistant to hydration therapy and has a high in-hospital mortality rate if the renal function of AKI patients does not react to an infusion load within a few days. Similarly, hydration therapy should be performed only in patients with reduced renal blood flow in CIN. If renal function does not recover within 2–3 days, it is classified as renal AKI and excessive hydration should be stopped. Furthermore, observational studies on seriously ill patients entering the ICU [287, 288] showed that excessive infusion with increased body fluid volumes did not suppress the progression of renal dysfunction and was an independent risk factor for in-hospital mortality. The CIN guideline 2012 emphasized that the hydration volume should be determined after carefully evaluating the body fluid volume. This revised guideline does not recommend hydration therapy unless body fluid volume is decreased.

### 10.3 CQ10-3 Dose low-dose dopamine therapy improve renal recovery after CIN onset ?

Answer:

We recommend against the use of low-dose dopamine for the treatment of CIN because it does not improve recovery from AKI.

**Level of Evidence: II/Grade of Recommendation: D**

**Rationale CQ10-3**

An RCT investigating the use of low-dose dopamine after the onset of CIN was the only one referenced in the CIN guideline 2012 [289]. In that study targeting post-PCI AKI patients and presumably including a large number of CIN patients, the peak SCr level and dialysis induction rate were significantly higher in the group treated with low-dose dopamine. Subsequently, Bellomo et al. [290] reported an RCT on low-dose dopamine treatment in AKI and showed that low-dose dopamine did not inhibit an increase in SCr levels and the induction of dialysis. In addition, Friedrich et al. reported that low-dose dopamine did not contribute to the prolongation of survival time or improvement in renal function in a meta-analysis of 61 studies examining the effect of low-dose dopamine for the treatment or prevention of AKI [291]. In a crossover study, Lauschke et al. [292] demonstrated that while pharmacologically low-dose dopamine reduced the renal resistive index (RI) in healthy subjects, it increased the RI and reduced renal blood flow in AKI patients. The AKI guideline 2016 [3] also recommended against using low-dose dopamine for preventive and therapeutic purposes in AKI. Thus, administration of low-dose dopamine after the onset of CIN is not recommended, because it does not inhibit the progression of renal dysfunction.

### 10.4 **CQ10-4** Does hANP treatment in CIN patients improve recovery from AKI?

Answer:

Administration of hANP aimed for AKI treatment after CIN onset is not recommended, because there is little evidence that it improves renal function or life prognosis after CIN onset.

**Level of evidence: I/Grade of Recommendation: D**

**Rationale CQ10-4**

In an RCT of critically ill patients with AKI, including patients with CIN, the dialysis-free survival for 21 days after treatment, percentage of patients undergoing dialysis by day 14, and all-cause mortality by day 21 did not differ significantly between patients who received high-dose hANP at 0.2 μg/kg/min for 24 h or those who received a placebo [293]. In an RCT of critically ill patients with oliguric AKI, the dialysis-free survival through day 21, percentage of patients undergoing dialysis by day 14, and mortality through day 60 did not differ significantly between patients receiving hANP and placebo [294]. Conversely, in a small RCT of patients with AKI associated with cardiac surgery who received a continuous infusion of low-dose hANP (50 ng/kg/min) or placebo immediately after the onset of AKI (SCr levels increased by 50% from baseline), there was no significant difference in the incidence of hypotensive episodes between the low-dose hANP and placebo groups, but the need for hemodialysis was significantly lower in the low-dose hANP group [295]. In a meta-analysis published in 2009, high-dose hANP did not significantly decrease mortality or the percentages of patients requiring hemodialysis and was associated with an increased incidence of hypotension [296]. Alternatively, low-dose hANP did not increase the incidence of hypotension or decrease the percentages of patients requiring hemodialysis. On the other hand, hANP is widely used in the treatment for heart failure, which is beyond the scope of this CQ.

Based on the above information, hANP administration after CIN onset has a limited effect on improving renal and life prognoses. Overall, hANP administration to treat AKI after the onset of CIN is not recommended. The AKI guideline 2016 also states that the use of low-dose hANP in the treatment of AKI is poorly supported [3]. However, low-dose hANP may be effective [1], and further investigation is expected.

### 10.5 **CQ10-5** Does acute blood purification therapy improve the outcome of renal function in patients with CIN?

Answer:

1. Acute blood purification therapy for improving renal function is not recommended, because there is no evidence demonstrating the improvement of renal function outcomes in patients with CIN.
2. Although not limited to AKI due to CIN, it is strongly recommended to perform acute blood purification therapy as a lifesaving strategy if the general condition becomes markedly poor due to body fluid volume, electrolyte, or acid–base balance abnormalities. The timing of the start of blood purification therapy should be determined according to the broader clinical context.

**Level of Evidence: I/Grade of Recommendation: B**

**Rationale CQ10-5**

No clinical trial related to acute blood purification therapy for improving the prognosis of renal function or mortality after CIN onset was found. Therefore, it is not recommended, as there is insufficient evidence that acute blood purification therapy improves renal function prognosis or mortality after CIN onset. Urgent blood purification therapy should be performed in patients with oliguric AKI, including CIN patients, in the case of severe life-threatening conditions. The KDIGO AKI guideline [1] states the following: “Initiate RRT emergently when life-threatening changes in fluid, electrolyte, and acid–base balance exist. (Not Graded)”

Although not limited to AKI due to CIN, it is strongly recommended to perform acute blood purification therapy as a lifesaving strategy if the general condition becomes markedly poor due to body fluid volume, electrolyte, or acid–base balance abnormalities.

Clinical trials on the timing of the start of blood purification therapy targeting only CIN patients have not reported, and a meta-analysis [297] showed that the effectiveness of early blood purification therapy for AKI including CIN was not clear. Therefore, the timing of the start of blood purification therapy should be determined in view of the patient’s overall clinical condition.
